# Amino acid transporter SLC38A5 is a tumor promoter and a novel therapeutic target for pancreatic cancer

**DOI:** 10.1038/s41598-023-43983-1

**Published:** 2023-10-06

**Authors:** Tyler Sniegowski, Devaraja Rajasekaran, Souad R. Sennoune, Sukumaran Sunitha, Fang Chen, Mohamed Fokar, Sudhir Kshirsagar, P. Hemachandra Reddy, Ksenija Korac, Mosharaf Mahmud Syed, Tanima Sharker, Vadivel Ganapathy, Yangzom D. Bhutia

**Affiliations:** 1 Department of Cell Biology and Biochemistry, Texas Tech University Health Sciences Center, Lubbock, TX 79430 USA; 2 Center for Biotechnology & Genomics, Texas Tech University, Lubbock, TX 79409 USA; 3 Department of Internal Medicine, Texas Tech University Health Sciences Center, Lubbock, TX 79430 USA

**Keywords:** Cancer, Pancreatic cancer

## Abstract

Pancreatic ductal adenocarcinoma (PDAC) cells have a great demand for nutrients in the form of sugars, amino acids, and lipids. Particularly, amino acids are critical for cancer growth and, as intermediates, connect glucose, lipid and nucleotide metabolism. PDAC cells meet these requirements by upregulating selective amino acid transporters. Here we show that SLC38A5 (SN2/SNAT5), a neutral amino acid transporter is highly upregulated and functional in PDAC cells. Using CRISPR/Cas9-mediated knockout of SLC38A5, we show its tumor promoting role in an in vitro cell line model as well as in a subcutaneous xenograft mouse model. Using metabolomics and RNA sequencing, we show significant reduction in many amino acid substrates of SLC38A5 as well as OXPHOS inactivation in response to SLC38A5 deletion. Experimental validation demonstrates inhibition of mTORC1, glycolysis and mitochondrial respiration in KO cells, suggesting a serious metabolic crisis associated with SLC38A5 deletion. Since many SLC38A5 substrates are activators of mTORC1 as well as TCA cycle intermediates/precursors, we speculate amino acid insufficiency as a possible link between SLC38A5 deletion and inactivation of mTORC1, glycolysis and mitochondrial respiration, and the underlying mechanism for PDAC attenuation. Overall, we show that SLC38A5 promotes PDAC, thereby identifying a novel, hitherto unknown, therapeutic target for PDAC.

## Introduction

Given the rapid proliferation capacity, PDAC cells have a great demand for nutrients in the form of sugars, amino acids, and lipids^1^. In particular, amino acids are critical for cancer growth and, as intermediates, connect glucose, lipid and nucleotide metabolism^2–4^. PDAC cells meet these requirements by upregulating selective amino acid transporters. Essential amino acids cannot be synthesized de novo and therefore need to be acquired from extracellular sources. Conversely, non-essential amino acids can be endogenously synthesized through precursors. However, because their synthetic capacity does not meet their increased demand, even for the non-essential amino acids, cancer cells predominantly rely on the extracellular sources, thereby indicating the importance of amino acid transporters for the cancer cells^5–8^.

Here we sought to study SLC38A5 (SN2/SNAT5) and its relevance to PDAC. SLC38A5 is a sodium-coupled neutral amino acid transporter which transports asparagine, histidine, glutamine, glycine, serine, and methionine^9,10^. It transports Na^+^ and the amino acid substrate in one direction by functionally coupling to the transfer of H^+^ in the opposite direction. Not much work has been conducted on SLC38A5 and its relevance to cancer except for two papers that talk about its role in PNET, and its relevance to macropinocytosis in triple-negative breast cancer^11,12^. Based on these literature evidences as well as on the functional features of SLC38A5, we wanted to investigate the role of SLC38A5 in PDAC growth and proliferation. These functional features include its ability to concentrate the amino acid substrates intracellularly while simultaneously removing H^+^ (cancer cells generate massive amounts of lactic acid, thus increasing H^+^ levels inside the cells) and preventing intracellular acidification, increasing the pH near the vicinity of the plasma membrane on the cytoplasmic side of the cell in the presence of amino acid substrates thus leading to induction of macropinocytosis (critical nutrient scavenging mechanism for PDAC), transport of glutamine for glutaminolysis (cancer cells are glutamine-addicted) and serine, glycine and methionine for one carbon metabolism required in purine/pyrimidine synthesis^6^.

Here we show that SLC38A5 is significantly upregulated in PDAC and the overexpression leads to poor overall survival in PDAC patients. Further characterizing the transporter, we show that SLC38A5 is not just overexpressed at the mRNA level but is also functional in PDAC cells. Using CRISPR/Cas9-mediated knockdown, we demonstrate the tumor promoting role of SLC38A5 in both cell lines and in athymic nude mice. Using metabolomics and RNA-sequencing, we show significant decrease in many of the amino acid substrates of SLC38A5 as well as inactivation of OXPHOS in response to SLC38A5 deletion. In addition to the already known substrates of SLC38A5, we show alanine, cysteine, threonine, valine, proline, phenylalanine, isoleucine and tyrosine as additional SLC38A5 substrates. Delving deeper into the mechanisms associated with PDAC attenuation in response to SLC38A5 deletion, we observe a serious metabolic crisis as evidenced by inhibition of glycolysis and mitochondrial respiration as well as inhibition of mTORC1 signaling pathway. Amino acid insufficiency is linked to inhibition of mTORC1 as well as suppression of critical glycolytic enzymes^13–15^. Moreover, amino acids function as TCA cycle intermediates/precursors^16^. Therefore, we speculate amino acid insufficiency as a possible link between SLC38A5 deletion and inhibition of mTORC1, glycolysis and mitochondrial respiration, thereby uncovering the underlying mechanism for the observed PDAC attenuation. Additionally, we show that SLC38A5 deletion does not lead to a compensatory upregulation of other amino acid transporters. Collectively, we show that SLC38A5 promotes PDAC and therefore could be a novel therapeutic target for PDAC.

## Results

### SLC38A5 is upregulated in PDAC and affects overall patient survival

**Figure 1:**
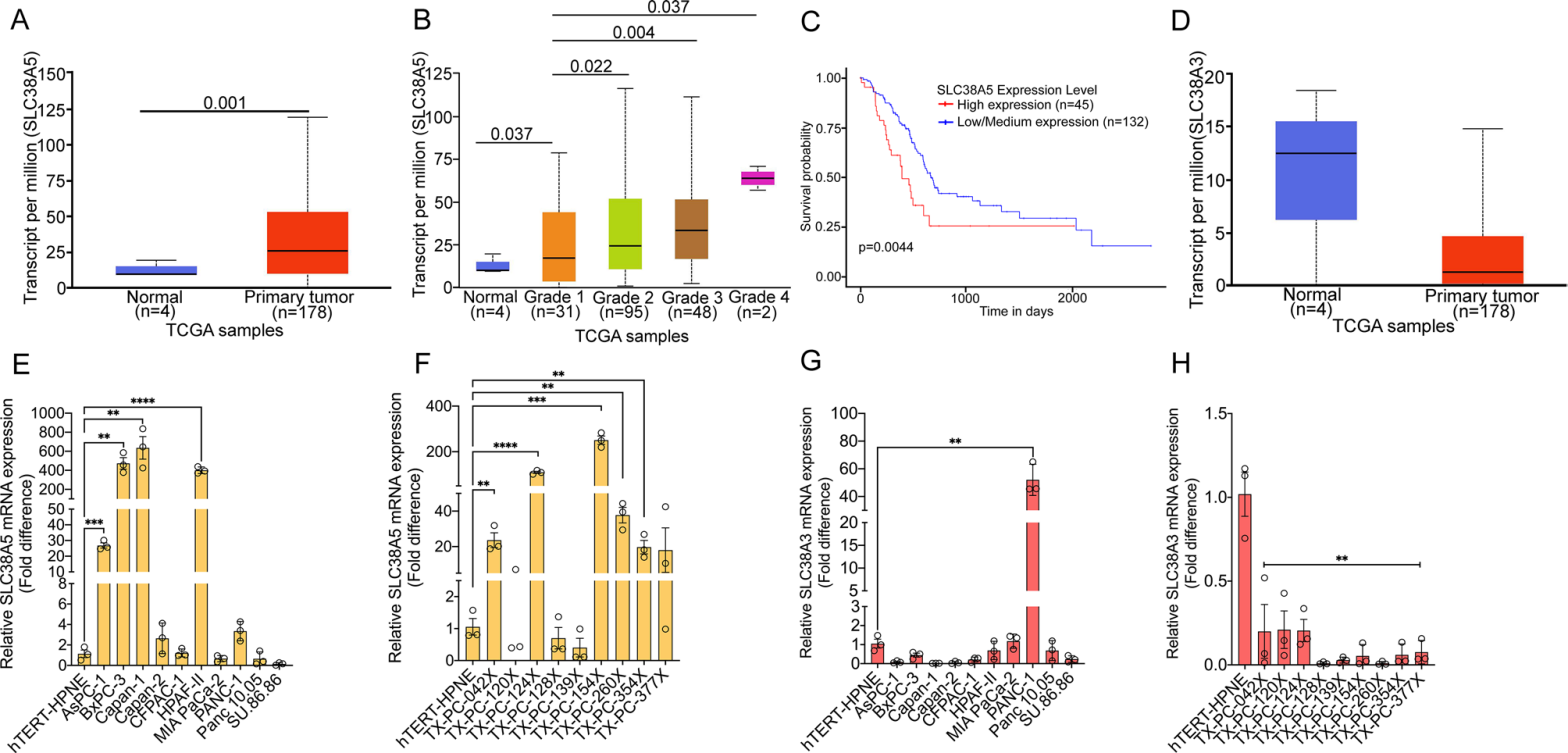
SLC38A5 is upregulated in PDAC and affects overall patient survival. (**A**) Box plot map showing TPM-normalized expression of SLC38A5 in normal tissues and pancreatic adenocarcinoma (PAAD). (**B**) Box plot map showing TPM normalized expression of SLC38A5 in normal tissues and pancreatic adenocarcinoma (PAAD) by tumor grade. (**C**) Kaplan–Meier plot showing survival probability between high and low SLC38A5 expression in pancreatic cancer. The result was generated from the online tool UALCAN (http://ualcan.path.uab.edu). Significance of survival impact is measured by log-rank test. (**D**) Box-plot map showing TPM-normalized expression of SLC38A3 in normal tissues and pancreatic adenocarcinoma (PAAD). Data derived from the TCGA database. (**E**) Real-time PCR showing relative mRNA expression of SLC38A5 in hTERT-HPNE (normal pancreatic epithelial cell line) and 10 PDAC cell lines. 18S was used as an endogenous control. (**F**) Real-time PCR showing relative SLC38A5 mRNA expression in hTERT-HPNE normal pancreatic cell line and 10 patient-derived xenografts (PDXs). (**G**) Real-time PCR showing relative mRNA expression of SLC38A3 in hTERT-HPNE (normal pancreatic epithelial cell line) and 10 PDAC cell lines. 18S was used as an endogenous control. (**D**) Real-time PCR showing relative SLC38A5 mRNA expression in hTERT-HPNE normal pancreatic cell line and 10 patient-derived xenografts (PDXs). Data are given as mean ± SEM. **p < 0.01, ***p < 0.001, ****p < 0.0001.

To study the role of SLC38A5 in PDAC growth and development, we first checked its expression profile in PDAC tumors vs normal pancreas using the web-based tool TCGA. SLC38A5 was found to be significantly upregulated at the transcript level in primary tumors of PDAC as opposed to normal pancreas (Fig. 1A). Additionally, we observed that SLC38A5 expression increased with a corresponding increase in the tumor grade (Fig. 1B). More interestingly, we found that PDAC patients with higher SLC38A5 expression had a significantly lower overall survival and vice versa (Fig. 1C). SLC38A3 expression (another system N amino acid transporter within the SLC38 gene family which is very similar to SLC38A5 in terms of substrate selectivity and functionality) remained unchanged (Fig. 1D). Using real-time PCR, we validated these online findings using PDAC cell lines (AsPC-1, BxPC-3, Capan-1, Capan-2, CFPAC-1, HPAF-II, MIA PaCa-2, PANC-1, Panc 10.05, and SU.86.86) and compared them to the levels found in hTERT-HPNE, a normal pancreatic ductal epithelial cell line. All the cell lines were cultured in complete medium. Four out of ten PDAC cell lines (AsPC-1, BxPC-3, Capan-1, and HPAF-II) showed a significant increase in SLC38A5 mRNA expression and the fold-increase ranged anywhere from 3 to ~ 600, relative to hTERT-HPNE (Fig. 1E). We further corroborated the cell lines data using nine PDAC patient-derived xenograft (PDX) samples wherein majority of the PDXs showed a significant increase in SLC38A5 expression wherein the fold-increase ranged from a minimum of 20 to a maximum of ~ 300, relative to hTERT-HPNE (Fig. 1F). SLC38A3 mRNA expression was downregulated in most of the PDAC cell lines (except PANC-1) and all PDXs tested (Fig. 1G,H). Taken together, the online data in conjunction with our laboratory validation clearly demonstrated that SLC38A5, and not SLC38A3, is significantly upregulated in PDAC and that the increased expression is associated with a poor overall patient survival.

### SLC38A5 is also upregulated in PDAC organoids

**Figure 2:**
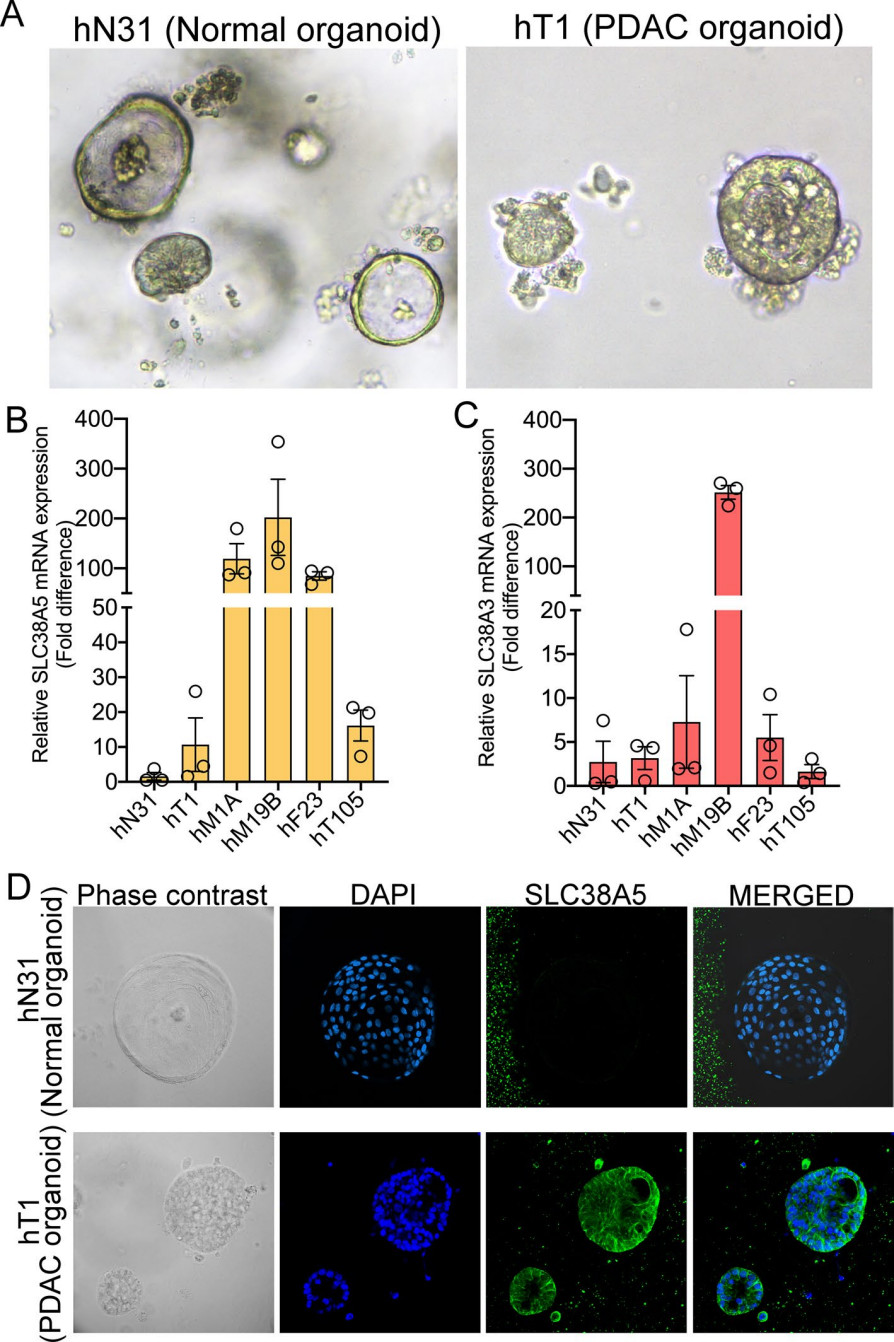
SLC38A5 is upregulated in PDAC organoids. (**A**) Phase-contrast image of hN31 normal pancreatic organoid and hT1 PDAC organoid at × 10 magnification. (**B,C**) Real-time PCR showing relative SLC38A5 and SLC38A3 mRNA expression in hN31 normal pancreatic organoid line and 5 PDAC organoid lines. Data are means ± SEM. (**D**) Immunocytochemical detection of SLC38A5 (green) in normal pancreatic organoid (hN31) and PDAC organoid (hT1). Nuclei stained with DAPI are blue. Magnification, × 60.

To further substantiate the cell lines and PDXs data, we checked SLC38A5 and SLC38A3 mRNA expression in human PDAC organoids that were cultured in complete medium. Organoids are three-dimensional models and therefore better recapitulate the human disease (Fig. 2A)^17–20^. Real-time PCR conducted in five human PDAC organoids (hT1, hM1A, hM19B, hF23, and hT105) and one normal human pancreatic organoid (hN31) demonstrated that SLC38A5 mRNA is upregulated in all the PDAC organoids tested when compared to the normal organoid (Fig. 2B). On the contrary, SLC38A3 mRNA was found to be comparable to that of hN31, except in hM19B (Fig. 2C). Furthermore, immunofluorescence data showed that the PDAC organoid (hT1) expressed SLC38A5 protein but the normal organoid (hN31) did not (Fig. 2D). Since hT1 was very easy to culture as opposed to other PDAC organoids, it formed the rationale for its selection for immunostaining. Collectively, the data acquired using different models demonstrate that SLC38A5 is indeed upregulated in PDAC whereas SLC38A3 is either unchanged or downregulated in PDAC.

### SLC38A5 is functional in PDAC cell lines

**Figure 3:**
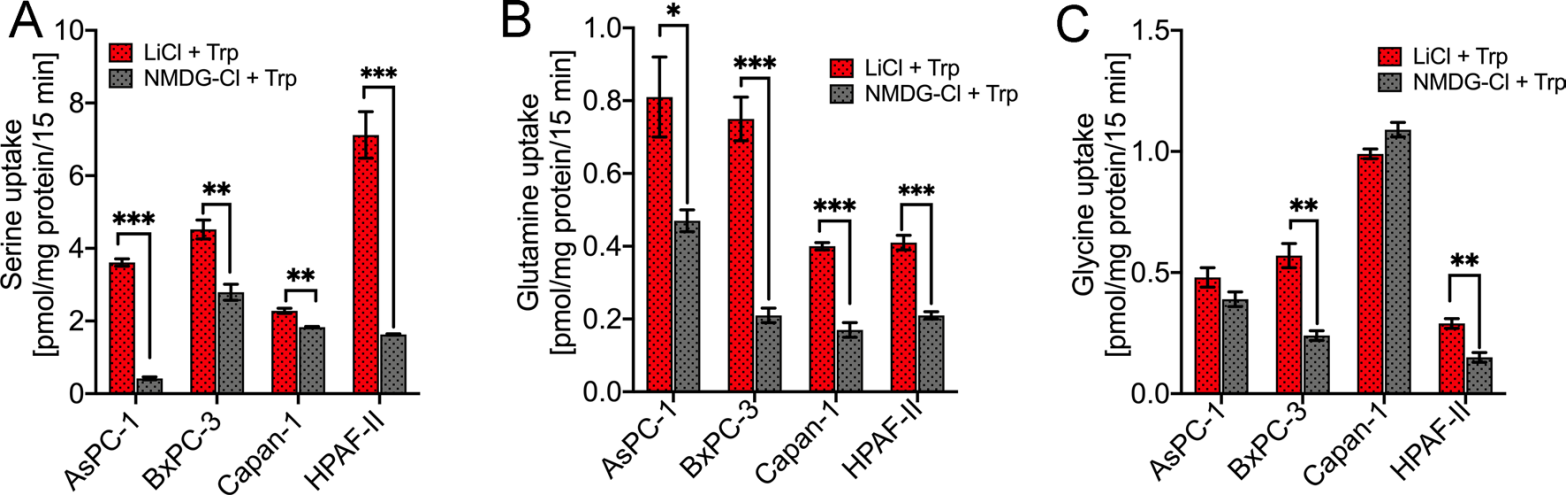
SLC38A5 is functional in PDAC cell lines. (**A–C**) SLC38A5-mediated ^3^H-serine uptake, ^3^H-glutamine uptake and ^3^H-glycine uptake in AsPC-1, BxPC-3, Capan-1, and HPAF-II PDAC cell lines. The uptake was conducted in LiCl or NMDG-Cl buffer containing 2.5 mM tryptophan at pH 8.5. Data are given as mean ± SEM. *p < 0.05, **p < 0.01, ***p < 0.001.

To test its functionality, we performed uptake studies in SLC38A5-positive AsPC-1, BxPC-3, Capan-1, and HPAF-II using serine, glutamine, and glycine as the radiolabeled substrates. The uptake was conducted in LiCl buffer as well as in NMDG-Cl buffer containing 5 mM tryptophan. The difference in the uptake between the LiCl buffer and NMDG-Cl buffer is considered the actual SLC38A5-mediated uptake. Based on the uptake data, it was obvious that the PDAC cell lines tested had functional SLC38A5, as demonstrated by SLC38A5-mediated uptake of serine, glutamine and glycine in the cell lines. While AsPC-1 had a robust SLC38A5-mediated serine uptake (88%) followed by glutamine (42%) and then glycine (17%), BxPC-3 on the other hand had a robust SLC38A5-mediated glutamine uptake (72%) followed by glycine (58%) and then serine (38%). In Capan-1, SLC38A5-mediated glutamine uptake was the maximum (57%) followed by serine (20%), however, no glycine uptake was observed in this cell line. Likewise, HPAF-II had a robust SLC38A5-mediated serine uptake (77%) followed by glutamine (49%) and glycine (49%) uptake (Fig. 3A–C). Taken together, the data indicated that SLC38A5 is not just overexpressed at the mRNA level, but is also functional in PDAC cell lines.

### shRNA-mediated knockdown of SLC38A5 indicates the tumor promoting ability of SLC38A5

To test the tumor promoting role of SLC38A5, shRNA-mediated knockdown of SLC38A5 was performed in HPAF-II cells. Of the five shRNAs tested, TRCN0000044003 demonstrated the most downregulation of SLC38A5 (~ 71%) as evident from the real time PCR data (Fig. S1A). Furthermore, the reduction in serine uptake in HPAF-II/pLKO.1-PURO/SLC38A5/shRNA (~ 82%) when compared to HPAF-II/pLKO.1-PURO clearly indicated that downregulation of SLC38A5 mRNA also translated to a reduction in its functionality (Fig. S1B). Colony formation assay demonstrated that SLC38A5 knockdown had some impact on the clonogenic ability of HPAF-II cells as evident from the lower number of colonies when compared to the control cells (Fig. S1C). In the mouse xenograft studies, the difference in the tumor volume between the control and knockout groups were not significantly different (Fig. S1D); however, the post-sacrifice tumor weight did show a significant downregulation in the knockout group (Fig. S1E). Body weight remained unchanged between both the groups (Fig. S1F). Overall, these data did indicate that SLC38A5 might be a tumor promoter, further alluding that a more efficient knockdown/knockout of SLC38A5 would enable us to better understand its role in tumor promotion in PDAC.

### CRISPR/Cas9-mediated knockout of SLC38A5 clearly demonstrated the tumor promoting role of SLC38A5 in both in vitro cell line models as well as in in vivo xenograft mouse models

**Figure 4:**
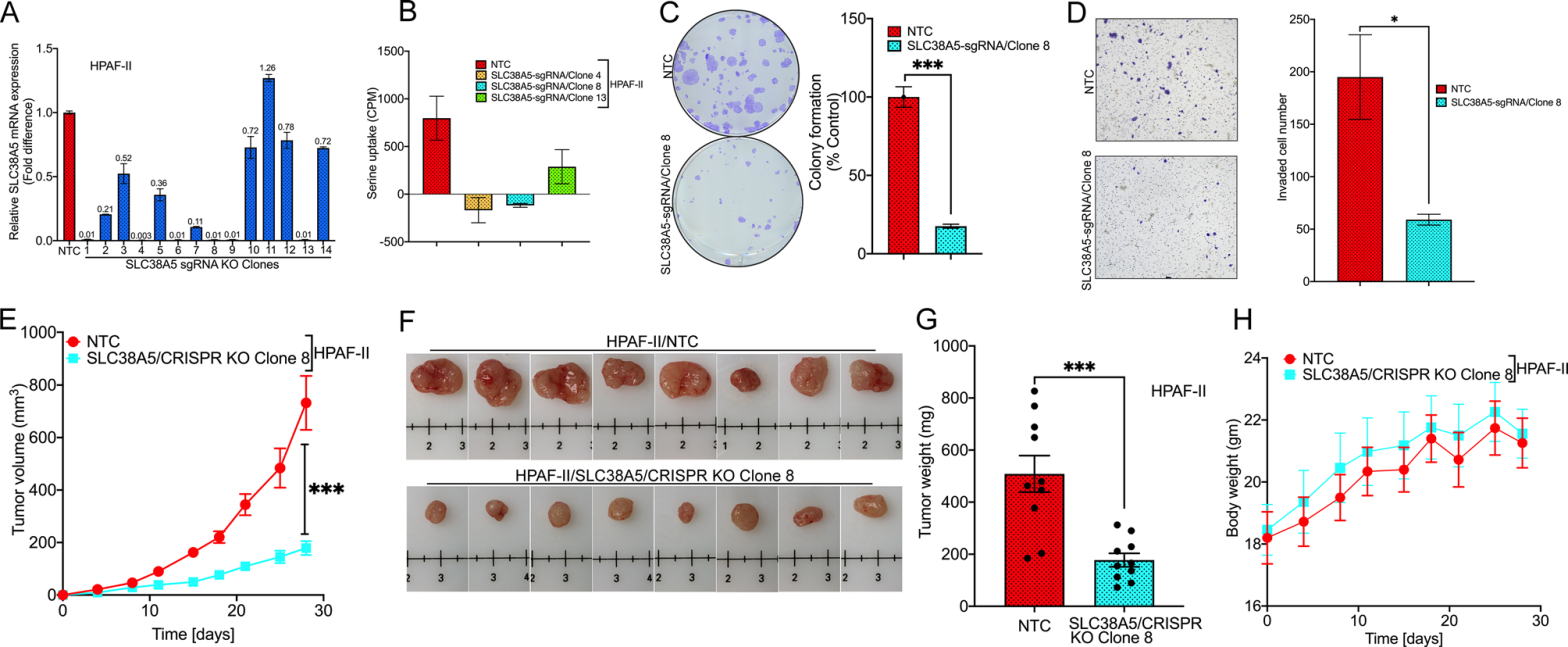
CRISPR/Cas9-mediated knockout of SLC38A5 and its tumor promoting role in vitro cell line models as well as in in vivo xenograft mouse models. (**A**) Real-time PCR of SLC38A5 mRNA expression in HPAF-II/NTC compared to HPAF-II/SLC38A5 CRISPR KO clones. (**B**) [^3^H]-Serine uptake in HPAF-II/NTC and three SLC38A5 CRISPR KO clones in LiCl buffer containing 2.5 mM tryptophan at pH 8.5. (**C**) Colony formation assay along with quantification in HPAF-II/NTC and HPAF-II/SLC38A5 KO clone 8. (**D**) Representative image (magnification × 10) of invasion assay along with cell count performed with HPAF-II/NTC and HPAF-II/SLC38A5 KO clone 8. (**E**) Tumor growth curves of HPAF-II/NTC and SLC38A5/KO clone 8 implanted as xenografts into athymic nude mice. (**F**) Representative photographs of harvested tumors from mice bearing HPAF-II/NTC and SLC38A5/KO clone 8. (**G**) End of study tumor weights between mice bearing HPAF-II/NTC and HPAF-II/SLC38A5 KO clone 8. (**H**) Body weights of mice xenografted with HPAF-II/NTC and HPAF-II/SLC38A5 KO clone 8. Data are given as mean ± SEM. *p < 0.05, ***p < 0.001.

In order to better understand the tumor promoting role of SLC38A5, CRISPR/Cas9 knockout was conducted. We began this process by transducing HPAF-II cells separately with the NTC lentivirus and the SLC38A5/KO sgRNA lentivirus. Using real-time PCR, we selected six (1, 4, 6, 8, 9, and 13) out of the fourteen selected clones that had an almost complete knockout of SLC38A5 (Fig. 4A). Furthermore, using radiolabeled serine uptake, we identified clones 4 and 8 to have complete loss of SLC38A5 functionality (Fig. 4B). Using clone 8 thereafter, colony formation assay and invasion assay was performed which clearly demonstrated that loss of SLC38A5 significantly impacted the clonogenic ability (~ 82% inhibition) (Fig. 4C) and the invasive property (~ 70% inhibition) (Fig. 4D) of HPAF-II cells. Likewise, in the in vivo mouse xenograft study, the group of mice injected with HPAF-II/SLC38A5/CRISPR KO cells had a significant reduction in tumor volume when compared to the group injected with HPAF-II/NTC cells (Fig. 4E). The tumor weight that was measured post-sacrifice was found to be significantly reduced in the CRISPR KO group (178 ± 29 mg) as opposed to the NTC group (509 ± 111 mg) (Fig. 4F,G). As expected, no significant change was observed in body weight between the NTC and the CRISPR KO groups (Fig. 4H). Taken together, the data clearly demonstrate the tumor-promoting role of SLC38A5.

### Metabolite profiling reveals significant downregulation of many amino acid substrates of SLC38A5 in response to SLC38A5 deletion

**Figure 5:**
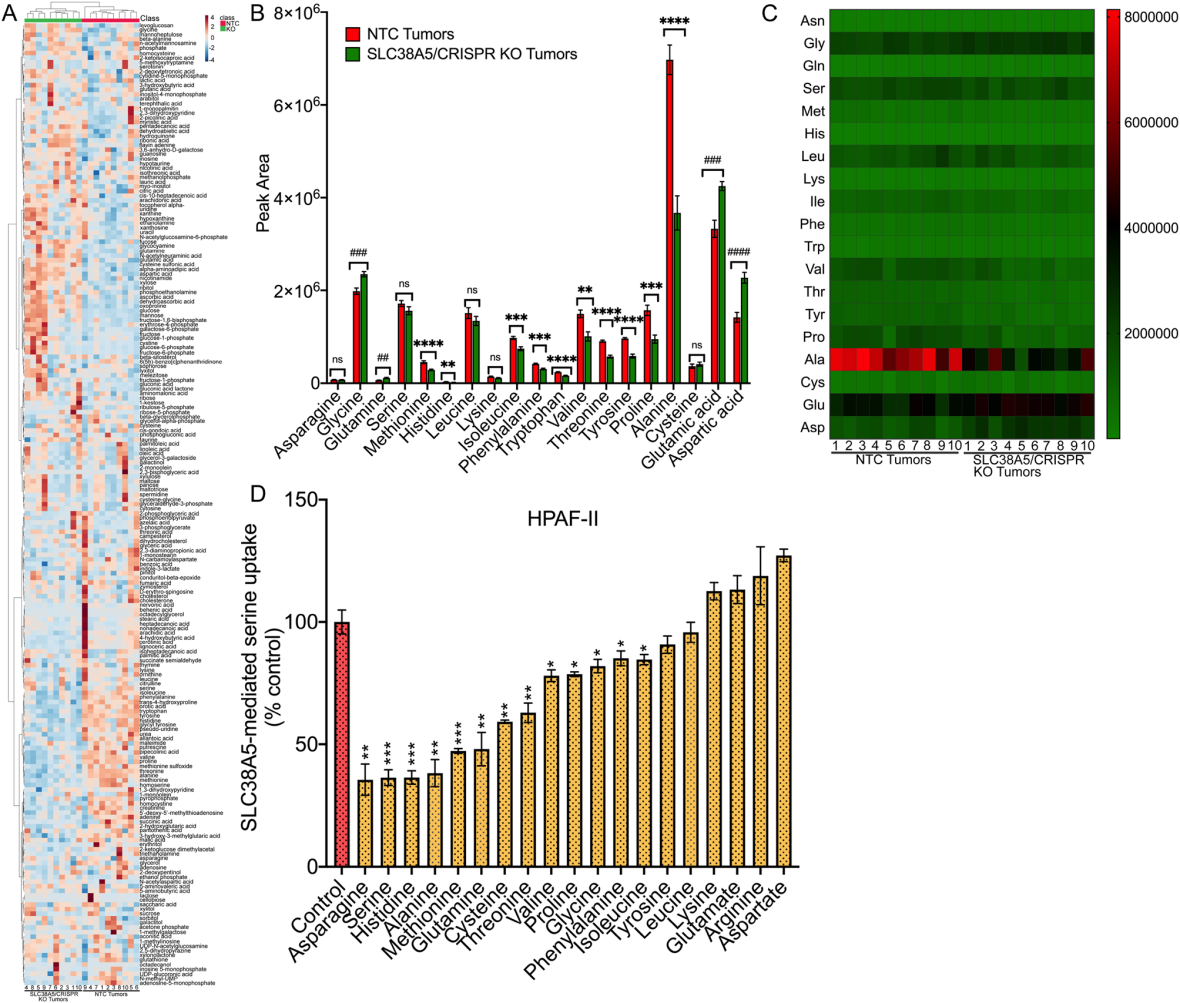
Metabolite profiling reveals significant downregulation of many amino acid substrates of SLC38A5 in response to SLC38A5 knockout. (**A**) Hierarchical clustering heatmap of metabolites from NTC tumors and SLC38A5/CRISPR KO tumor as analyzed by MetaboAnalyst 4.0. (**B**) Amino acid peaks between the NTC tumors and the SLC38A5/CRIPSR KO tumors from the metabolomic analysis. (**C**) Heatmap representation of the amino acid peak between the NTC tumors and the SLC38A5/CRIPSR KO tumors from the metabolomic analysis. (**D**) [^3^H]-Serine uptake in HPAF-II cells showing amino acid substrate selectivity. The uptake was conducted using LiCl buffer containing 5 mM tryptophan at pH 8.5, either in the presence or absence of 5 mM of various amino acids. Uptake measured in NMDG-Cl buffer was subtracted from the uptake measured in the presence of LiCl (with or without the competing amino acid) in order to calculate the Li^+^-coupled uptake. Data are given as mean ± SEM. *NS* non-significant, *p < 0.05, **p < 0.01, ***p < 0.001, ****p < 0.0001, ^##^*p* < 0.01, ^###^*p* < 0.001, ^####^*p* < 0.0001.

To understand how SLC38A5 loss attenuates tumor growth in athymic nude mice, we wanted to check the amino acid profile between the control and KO tumors. This could provide us clues to understand tumor attenuation mechanisms associated with SLC38A5 knockout. Amino acids are known activators of mTORC1, a serine/threonine kinase signaling pathway that is significantly activated in cancer^21–26^. In the past, only leucine, arginine, and methionine were known to activate mTORC1, however, with recent discoveries, it is known that other amino acids like alanine, asparagine, glutamine, histidine, serine, threonine, and valine also activate mTORC1^27^. Interestingly, most of these amino acids (asparagine, glutamine, histidine, methionine and serine) are SLC38A5 substrates. Therefore, we hypothesized that SLC38A5 loss would significantly impact the levels of these amino acids in the KO tumors, leading to mTORC1 inactivation, ultimately leading to an overall reduction in tumor growth. To test that, metabolomics was performed in xenograft tumors (n = 10) from HPAF-II/NTC to HPAF-II/SLC38A5 CRISPR KO. The hierarchical clustered heatmap of the metabolomic data did show distinct clustering of several metabolites either in the NTC or the KO tumors, suggesting a difference in the metabolite profile between the two groups (Fig. 5A). We then focused on the amino acid profile and interestingly, found several amino acids to be significantly reduced, some of them to be upregulated and while the rest remained unchanged in the KO tumors as opposed to the NTC tumors. Focusing on SLC38A5 substrates, we found asparagine and serine levels to be unchanged, glycine and glutamine levels to be upregulated while that of methionine and histidine to be significantly downregulated. More interestingly, other amino acids like isoleucine, phenylalanine, tryptophan, valine, threonine, tyrosine, proline, and alanine were significantly downregulated whereas aspartic acid, and glutamic acid were significantly upregulated. On the other hand, leucine, lysine and cysteine remained unchanged (Fig. 5B,C). Because we saw downregulation in many other amino acids apart from the known substrates of SLC38A5, we wanted to test if these downregulated amino acids are also SLC38A5 substrates. For that, radiolabeled serine uptake was conducted in HPAF-II to check for the substrate selectivity, either in the presence or absence of 5 mM concentration of various amino acids. As expected, we saw ~ 64% inhibition of ^3^H-serine uptake with asparagine, serine, and histidine, ~ 52% inhibition with methionine and glutamine, and ~ 18% inhibition with glycine, which are all known substrates of SLC38A5 (Fig. 5D). More interestingly, we found significant inhibition of ^3^H-serine uptake with alanine (62%), which was comparable to that of asparagine, serine, and histidine. Other amino acids that inhibited ^3^H-serine uptake include cysteine (41%), threonine (37%), valine and proline (21%), phenylalanine (15%), isoleucine and tyrosine (11%). Amino acids that did not inhibit ^3^H-serine uptake include tyrosine, leucine, lysine, glutamate, arginine and aspartate. Taken together, the data demonstrates that SLC38A5 KO led to a significant reduction in many amino acids and interestingly, most of them are transportable substrates of SLC38A5. Furthermore, it was interesting to observe that five out of the eight amino acids substrates that were downregulated are also mTORC1 activators, suggesting mTORC1 inhibition as one of the possible mechanisms of tumor attenuation in response to SLC38A5 knockout.

### SLC38A5 knockout inhibits mTORC1 signaling pathway and is not associated with a compensatory upregulation of other amino acid transporters

**Figure 6:**
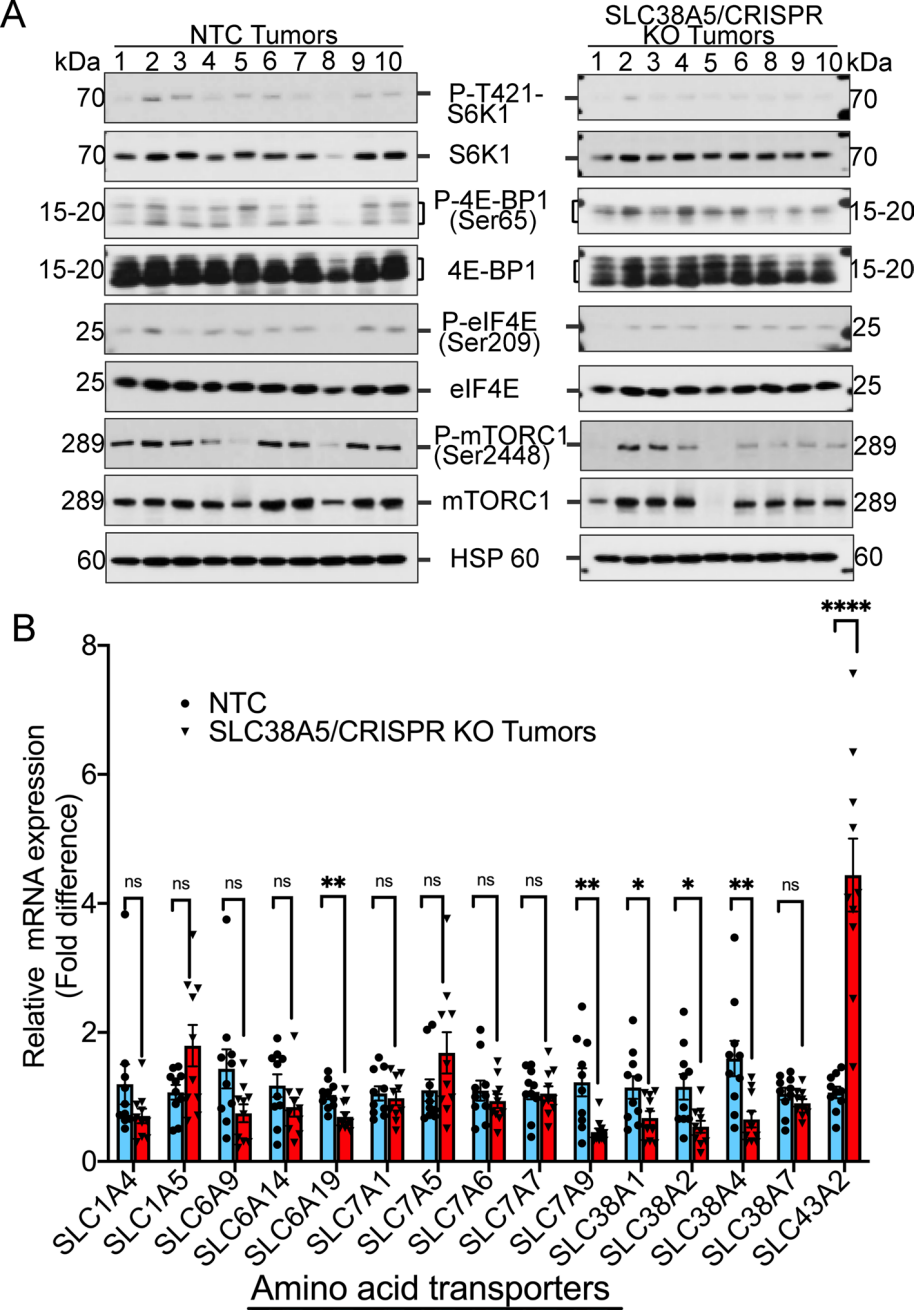
SLC38A5 knockout inhibits mTORC1 signaling pathway and is not associated with a compensatory upregulation of other amino acid transporters. (**A**) Western blotting showing evaluation of mTORC1 and its downstream targets (both phosphorylated and total) in NTC tumors and SLC38A5/CRISPR KO tumors. HSP 60 was used as an endogenous control. (**B**) Real-time PCR showing mRNA expression of 15 amino acid transporters in NTC tumors and SLC38A5/CRISPR KO tumors. Data are given as mean ± SEM. *NS* non-significant, *p < 0.05, **p < 0.01, ****p < 0.0001.

The fact that SLC38A5 knockout led to a significant change in the amino acid profile and because many of the downregulated amino acids were the ones that activate mTORC1, we wanted to check whether this change actually resulted into mTORC1 pathway inhibition. To test that, western blotting was performed in 10 of the NTC tumors and 9 of the SLC38A5/CRISPR KO tumors. As a read-out for activation of mTORC1 pathway, we checked for the phosphorylation status of p70 S6K1 and 4EBP1. Increased phosphorylation of these downstream effectors of mTORC1 would indicate activation of the pathway^28,29^. SLC38A5 knockout inhibited phosphorylation of both S6K1 and 4EBP1 indicating inhibition of mTORC1 signaling pathway (Fig. 6A). To further corroborate this data, we also checked the phosphorylation status of eIF4E. When mTORC1 is activated, it can phosphorylate 4EBP1, causing it to dissociate from eIF4E which then enables interaction of eIF4E with eIF4G and eIF4A, thus initiating polypeptide synthesis. However, when mTORC1 is inhibited, 4EBP1 can bind competitively with eIF4E, inhibit its interaction with eIF4G and thus inhibit translation^30^. Western blotting demonstrated that knockout of SLC38A5 inhibited eIF4E phosphorylation, further supporting the fact that mRNA translation was inhibited. Furthermore, mTORC1 phosphorylation was also inhibited. Total protein for all the targets remained mostly unchanged (Fig. 6A). Together, the data clearly indicated that SLC38A5 knockout inhibited mTORC1 signaling pathway, thereby attenuating PDAC growth.

To test whether SLC38A5 knockout led to a compensatory upregulation of other amino acid transporters (AATs), we performed real-time PCR in 10 NTC tumors and 10 SLC38A5/CRISPR KO tumors to check for the mRNA expression of 15 AATs. The AATs tested included neutral amino acid transporters (SLC1A4, SLC1A5, SLC6A19, SLC7A5, SLC38A1, SLC38A2, SLC38A3, SLC38A4 and SLC43A2), neutral and cationic AATs (SLC6A14, SLC7A6, SLC7A7, and SLC7A9), and neutral, cationic and anionic AAT (SLC38A7)^7,8,31,32^. These AATs were selected based on the fact that they also transport neutral amino acids including the ones that are transportable substrates for SLC38A5. Our rationale was that when SLC38A5 is deleted, the cancer cells would find a way to upregulate any of these transporter/transporters to compensate for the loss. Contrary to our belief, we found the mRNA expression of 5 of the AATs (SLC6A19, SLC7A9, SLC38A1, SLC38A2, SLC38A4) tested to be significantly downregulated with the exception of SLC43A2, whose expression was found to be significantly upregulated (Fig. 6B). More interestingly, the expression pattern of the remaining AATs was either unchanged or exhibited a downregulation trend. SLC38A3 expression was undetectable in the KO tumors (data not shown). Therefore, it was interesting to observe that SLC38A5 knockout either did not change or reduced the expression of the majority of the AATs tested, with the exception of SLC43A2. SLC43A2, also known as LAT4 transports methionine, isoleucine, leucine and phenylalanine^33^. Overall, the data indicate that SLC38A5 knockout inhibits mTORC1 signaling pathway and is not associated with a compensatory upregulation of other AATs, implicating these as the plausible reason for the significant attenuation of tumor growth in the athymic nude mice.

### RNA sequencing reveals significant impact on oxidative phosphorylation following SLC38A5 knockout

**Figure 7:**
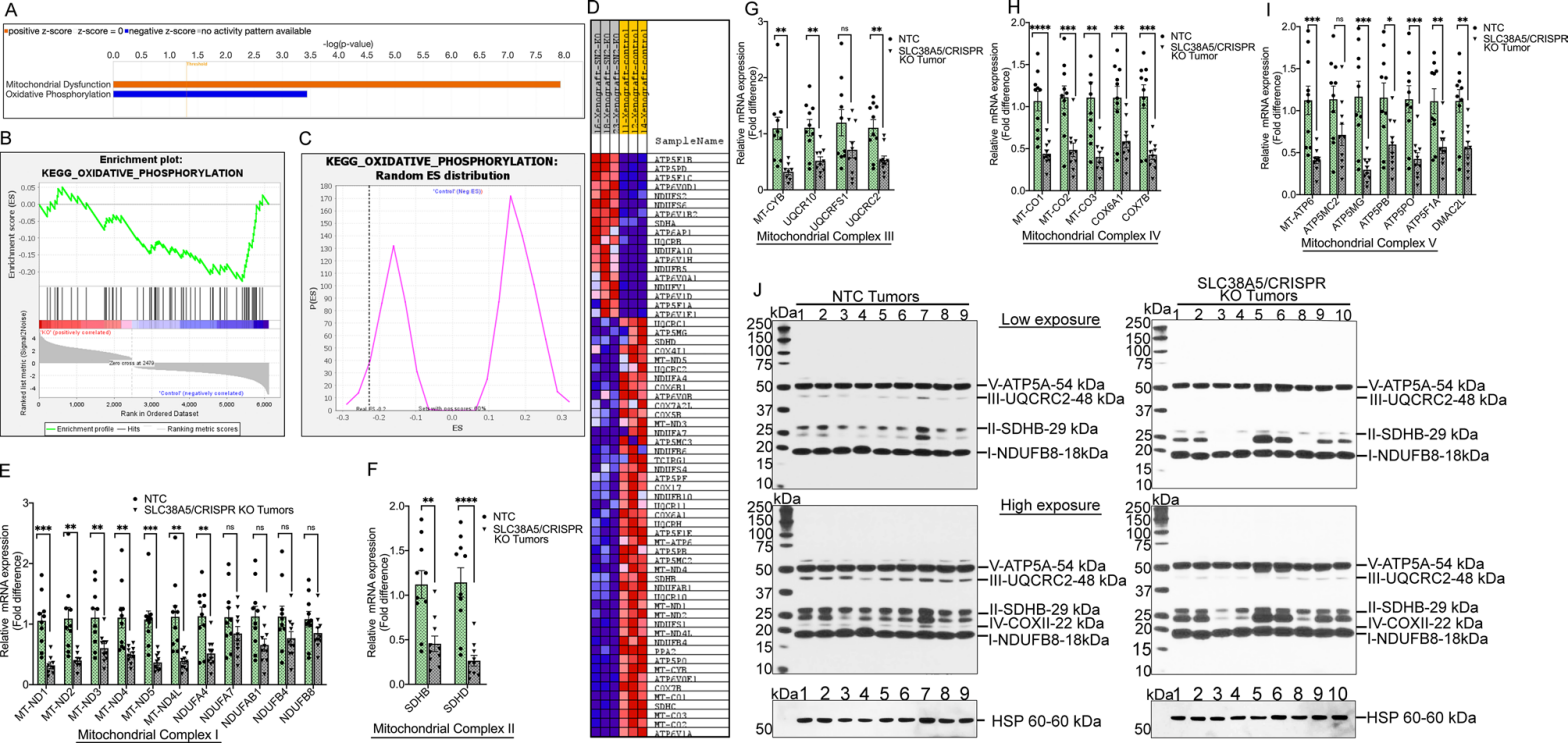
RNA sequencing reveals significant impact on oxidative phosphorylation following SLC38A5 knockout. (**A**) Ingenuity Pathway Analysis plot for the top 2 canonical pathways out of the 20 enriched pathways as analyzed by the IPA software. Positive z-score shown in orange identifies activated pathway and negative z-score in blue color specifies inhibited pathways after CRISPR-mediated knockout of SLC38A5. (**B,C**) Gene set enrichment analysis (GSEA) plot and random ES distribution plot showing changes in oxidative phosphorylation pathway following CRISPR-mediated knockout of SLC38A5. (**D**) Heatmap of genes associated with oxidative phosphorylation, either upregulated or downregulated in SN2-KO (SLC38A5/KO) tumors when compared to control tumors. (**E–I**) Real-time PCR showing relative mRNA expression of genes related to mitochondrial complex I, II, III, IV, and V. (**J**) Western blotting showing protein levels from each of the five mitochondrial respiratory complexes i.e., NDUFB8 (Complex I), SDHB (Complex II), UQCRC2 V (Complex III), COXII (Complex IV), and ATP5A (Complex V). HSP 60 was used as the endogenous control. Data are given as mean ± SEM. *NS* non-significant, *p < 0.05, **p < 0.01, ***p < 0.001, ****p < 0.0001.

Having identified mTORC1 signaling pathway inactivation as a mechanism for tumor attenuation in response to SLC38A5 knockout, we were curious to identify additional pathways or targets that could function either independently or in conjunction with mTORC1 pathway, to reduce tumor growth in PDAC. To test that, we performed RNA sequencing using the HPAF-II/NTC (n = 3) and HPAF-II/SLC38A5-CRISPR KO (n = 3) xenograft tumors. Using the IPA software, we checked the canonical pathways that are likely activated or inhibited in response to SLC38A5 knockout. A total of 20 enriched canonical pathways were identified by applying the − log (p-value) > 2 threshold (Fig. S2). Many signaling pathways that are associated with tumor growth and promotion were significantly inhibited; however, the most striking pathway was the mitochondrial dysfunction, and the OXPHOS inactivation in the KO tumors (Fig. 7A). Mitochondrial dysfunction had a − log (p-value) of 7.935 and a Z-score of 3.938. Likewise, for OXPHOS the − log (p-value) was 3.44 and the Z-score was − 4.315. Focusing more on the OXPHOS, KEGG analysis was performed. The gene set enrichment analyses (GSEA), random ES distribution and the heat map clearly exemplifies the genes that are deregulated in OXPHOS in response to SLC38A5 KO (Fig. 7B–D). Thereafter, we wanted to experimentally validate that OXPHOS was in fact significantly impacted in SLC38A5 KO tumors, which could in turn be the other mechanism in addition to suppression of mTORC1 signaling pathway contributing to PDAC attenuation. For that, real-time PCR was conducted in NTC and SLC38A5/KO tumors (n = 10) to test for the mitochondrial genes that are specific for mitochondrial respiratory complex I (NADH: ubiquinone oxidoreductase genes), complex II (SDH: succinate dehydrogenase subunits), complex III (ubiquinol-cytochrome C reductase complex subunits), complex IV (cytochrome C oxidase genes) and Complex V (ATP synthase genes). The lists of genes tested were selected based on the RNA-sequencing data. It was very interesting to note that all the genes tested in all the 5 mitochondrial complexes were significantly downregulated (Fig. 7E–I). To test whether this reduction in mRNA expression also translated into proteins, western blotting was conducted. Using the OXPHOS antibodies cocktail, we monitored the level of one protein from each of the five mitochondrial respiratory complexes i.e., NDUFB8 (Complex I), SDHB (Complex II), UQCRC2 V (Complex III), COXII (Complex IV), and ATP5A (Complex V). Interestingly, significant reduction was seen in the protein level of SDHB, UQCRC2, and COXII that belong to mitochondrial respiratory complex II, III, and IV, respectively. NDYFB8 and ATP5A from complex I and V remained unchanged (Fig. 7J). Taken together, the data clearly demonstrate that loss of SLC38A5 significantly impacts the expression status of mitochondrial genes and proteins that are specific for OXPHOS. This could have affected the functional efficiency of the OXPHOS metabolic pathway, resulting into reduced ATP output and ultimately attenuation of PDAC growth.

### SLC38A5 knockout significantly decreases both mitochondrial respiration as well as glucose metabolism in PDAC cells

**Figure 8:**
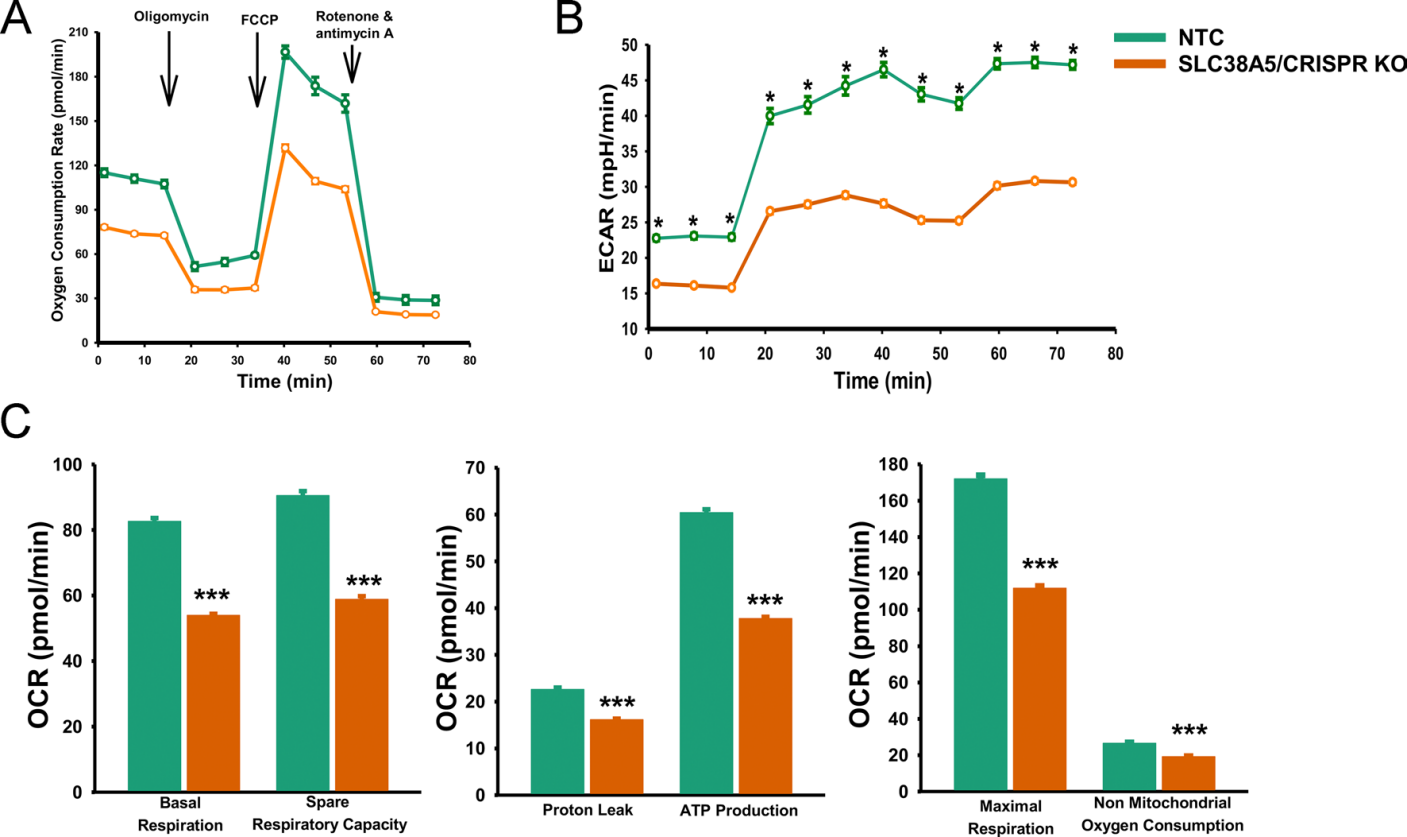
Mitochondrial bioenergetic state in NTC cells compared to SLC38A5/CRISPR KO cells. (**A**) Kinetic OCR (oxygen consumption rate) response of NTC and SLC38A5/CRISPR KO cells to oligomycin (2 µM) to determine ATP coupled respiration, FCCP (1 µM) to determine maximal respiration, and rotenone and antimycin A (0.5 µM) to define spare respiratory capacity. Cells were plated at 2 × 10^4^/well overnight prior to the assays. The OCR values were reported to pmol/min. Each data point represents mean ± SEM. (**B**) Kinetic ECAR (Extracellular Acidification Rate) response of NTC and SLC38A5/CRISPR KO cells to oligomycin (2 µM), FCCP (1 µM), and rotenone and antimycin A (0.5 µM). Cells were plated at 2 × 10^4^/well overnight prior to the assays. The ECAR values were reported as mpH/min. Each data point represents mean ± SEM. *p < 0.001. (**C**) Graphs represent calculated basal respiration, spare respiratory capacity, proton leak-linked respiration, ATP production, maximal mitochondrial respiration and non-mitochondrial oxygen consumption. OCR values are expressed as pmol/min. Graphs represent the mean ± SEM. ***p < 0.001.

The classical definition of mitochondrial dysfunction is the inability of mitochondria to sustain ATP synthesis sufficient to satisfy cellular demands^34^. Though it is known that mitochondrial dysfunction exhibits many faces, causes, and consequences, at least in the context of SLC38A5 knockout, OXPHOS seems to be significantly impacted. Therefore, we wanted to measure the oxygen consumption rate (OCR) and extracellular acidification rate (ECAR) as a readout to investigate the effects of SLC38A5 knockout on the metabolic phenotype of PDAC cells. To test that, HPAF-II/NTC cells and HPAF-II/SLC38A5/CRISPR-KO cells were used. Cancer cells are known to shift their energetic metabolism from mitochondrial respiration to aerobic glycolysis^35,36^. Since OXPHOS was impacted in SLC38A5 KO cells, we hypothesized that there will be a reciprocal relationship between OCR and ECAR. Contrary to our belief, we found both OCR and ECAR to be significantly decreased in SLC38A5/KO cells as opposed to the NTC cells (Fig. 8). Reduction in OCR was indicated by decrease in basal OCR and spare respiratory capacity, decreased ATP production and inhibition of maximal respiration. Likewise, reduction in ECAR was demonstrated by reduced ECAR under basal glycolysis, lower percentage of ECAR from basal glycolysis, and reduced ECAR of compensatory glycolysis. Taken together, the data suggest that SLC38A5 KO leads to a profound metabolic crisis in the KO cells, impacting both glycolysis and mitochondrial respiration, ultimately leading to attenuation of PDAC growth.

## Discussion

We demonstrate for the first time that SLC38A5 is critical for PDAC growth and proliferation. Literature evidences show the proliferative role of SLC38A5 viz., promoting retinal neovascularization to upregulation in inflammatory conditions of the intestine as well as proliferation of glucagon-secreting α-cells^37–40^. From a cancer perspective, SLC38A5 induces a sub-population of α-cells to induce PNET^11^ and recently, we have shown the relevance of SLC38A5 in inducing macropinocytosis in TNBC^12^. These literature evidences, substantiated with our own findings demonstrate SLC38A5 as a tumor promoter.

SLC38A5 KO significantly attenuated PDAC growth. While that was an interesting finding, the metabolomics data showing significant reduction in many amino acids, both transportable as well as non-transportable substrates of SLC38A5 was baffling. While many SLC38A5 substrates were downregulated, glutamine and glycine levels were upregulated. Since many AATs identify glutamine and glycine as their substrates, it is possible that in spite of SLC38A5 KO, PDAC cells are able to maintain their concentration, in fact at a higher level to compensate for the loss of amino acids. We also demonstrate that apart from the known substrates, SLC38A5 also identifies alanine, threonine, cysteine, valine, proline, phenylalanine, and isoleucine as additional substrates, clearly explaining the profound reduction in amino acid levels in the KO tumors.

Furthermore, we show mTORC1 inhibition as a consequence of SLC38A5 KO. In the past, only three amino acids viz., leucine, arginine, and methionine were known to activate mTORC1; however, with recent discoveries, it is known that other amino acids like alanine, asparagine, glutamine, histidine, serine, threonine, and valine also activate mTORC1. Since many of these amino acids are SLC38A5 substrates and since their levels were reduced in the KO tumors, it makes sense as to why mTORC1 pathway was inhibited in the KO tumor samples. Furthermore, our results showing lack of compensatory upregulation by other AATs seems important, though perplexing. There could be myriad of reasons; however, we speculate inhibition of mTORC1 signaling pathway associated with SLC38A5 KO as a possible explanation. mTORC1 signaling pathway is regulated by amino acids and therefore it is possible that the synthesis of many of these transporters could be regulated by mTORC1. However, these possibilities require further validation. Nevertheless, this is an interesting finding suggesting that KO of SLC38A5 alone should be able to diminish the expression of these AATs simultaneously. Not much is known about these AATs and their relevance to PDAC except SLC38A2, which is activated in PDAC cells to fuel their alanine demands^41^. On the other hand, SLC43A2/LAT4 was upregulated in the KO tumors. SLC43A2 transports methionine and it has been specifically shown that upregulation of this transporter in tumor cells depletes methionine in the tumor microenvironment leading to T-cell depletion and tumor evasion^33^. Therefore, this could be an effort by the KO tumors to compensate for the loss of SLC38A5. However, the fact that SLC43A2 substrate (methionine, phenylalanine, isoleucine, and leucine) levels were still lower, suggests that upregulating SLC43A2 itself is not enough to compensate for the loss of SLC38A5.

SLC38A5 KO significantly downregulates OXPHOS-associated genes and proteins. Functionally, we show inhibition of glycolysis and mitochondrial respiration indicating that SLC38A5 KO leads to a serious metabolic crisis. Could deficiency of amino acids lead to these consequences? Amino acids are used for protein synthesis or oxidized as energy sources. They serve as precursors of many important metabolites that regulate gene expression, post-translational modifications of proteins and cell fate. Literature evidence shows that amino acid deficiency lowers mitochondrial membrane potential and leads to early onset of mitochondrial respiratory quiescence during oogenesis in flies. The lack of amino acids in the cell is sensed by general control non-derepressible 2 (GCN2), which has a high affinity to uncharged tRNAs. Upon binding to an uncharged tRNA, GCN2 undergoes a conformational change, gets activated and inhibits phosphorylation of eukaryotic translation initiation factor 2α (eIF2α), and prevents translation initiation under amino acid deficiency. Processes such as glucose uptake, nucleic acid synthesis and, of course, protein synthesis are rapidly compromised due to amino acid deficiency. Interestingly, studies have shown that deficiency of essential amino acids inhibit phosphofructokinase (PFK1) enzyme by uncharged tRNA, leading to accumulation of glucose-6-phosphate, a potent inhibitor of hexokinase which promotes glucose uptake by phosphorylation^14,15,42–45^. Since SLC38A5 KO leads to a significant reduction in the essential amino acids level, this could be the possible explanation for reduction in OXPHOS protein expression as well as inhibition of glycolysis. Literature evidence has also shown that loss of SLC38A2 leads to profound compartmentalized metabolic crisis wherein both glycolysis and mitochondrial respiration is inhibited in the PDAC cells^41^. We see similar effects except alanine is one among many amino acids inhibited in response to SLC38A5 KO. Therefore, we speculate amino acid deficiency as the possible link connecting mTORC1 inhibition as well as inhibition of glycolysis and mitochondrial respiration as mechanisms resulting in PDAC attenuation post SLC38A5 KO. We further speculate that SLC38A5 KO inhibited macropinocytosis further contributing to amino acid loss, thereby attenuating PDAC growth. These speculations, however, require validation.

Taken together, we show that SLC38A5 is significantly upregulated in PDAC and its knockout significantly attenuates tumor growth. Mechanistically, we show SLC38A5 KO significantly inhibits mTORC1 signaling pathway as well as inactivates glycolysis and mitochondrial respiration. Our laboratory has also identified SLC6A14, another AAT as a novel PDAC target^46,47^. Therefore, SLC38A5 could be equally invaluable as SLC6A14 in targeting PDAC, however, their relative contribution to PDAC needs further validation.

## Materials and methods

### Materials

[2,3-^3^H]-l-Serine (specific radioactivity, > 5 Ci/mmol) was purchased from Moravek, Inc. (Brea, CA, USA). [2-^3^H]-Glycine (specific radioactivity, 42.4 Ci/mmol), and [3,4-^3^H]-glutamine (specific radioactivity, 50.5 Ci/mmol) were purchased from PerkinElmer Corp (Waltham, MA, USA). Primary antibody SLC38A5 rabbit polyclonal (ab72717) was from Abcam (Waltham, Boston, U.S.A.). Secondary antibody goat anti-rabbit IgG (H+L) cross-adsorbed secondary antibody, Alexa Fluor™ 488 was from Invitrogen (USA, Cat # A-11008).

### Cell culture

hTERT-HPNE, a human normal pancreatic ductal epithelial cell line, as well as human pancreatic cancer cell lines AsPC-1, BxPC-3, Capan-1, Capan-2, CFPAC-1, HPAF-II, MIA PaCa-2, PANC-1, Panc 10.05, and SU.86.86 were obtained from ATCC. The cell lines were used within 10–20 passages for all experiments. ATCC has done morphological, cytogenetic and DNA profile analyses for characterization of these cell lines. hTERT-HPNE cells were maintained in 75% Dulbecco’s Modified Eagle’s Medium (DMEM) without glucose plus 25% Medium M3 Base with the following additives: 5% FBS (fetal bovine serum), 5.5 mM d-glucose, 10 ng/ml human recombinant epidermal growth factor, and 750 ng/ml puromycin. The remaining cell lines were maintained in the respective media suggested by the ATCC (DMEM, RPMI-1640, Eagle’s Minimum Essential Medium, and Iscove’s DMEM) supplemented with 10% FBS, except for Panc 10.05 that required 15% FBS. All media contained 100 U/ml penicillin, and 100 U/ml streptomycin. All media for the above cell lines were purchased from Corning (Manassas, VA, U.S.A.) whereas FBS was from Gibco (U.S.A. Cat #26140079), and plasticware for cell culture was obtained from Corning LifeSciences (Manassas, VA, U.S.A.). Cells were cultured at 37 °C in a humidified atmosphere containing 5% CO2. All these cell lines have been routinely tested for mycoplasma contamination using the Universal Mycoplasma Detection Kit obtained from ATCC.

### Organoid culture

Human normal pancreatic organoid (hN31) and human PDAC organoids (hT1, hM1A, hM19B, hF23, and hT105) were procured from the Tuveson lab with a completed Materials Transfer Agreement. These organoids were procured from Tuveson lab and all materials and methods for culturing the organoids were used following the Tuveson organoid protocols (https://tuvesonlab.labsites.cshl.edu/wp~content/uploads/sites/49/2018/06/20170523_OrganoidProtocols.pdf). Briefly, organoids were suspended in matrigel and placed in 75 μl aliquots into a 24 well plate. These matrigel domes were allowed to harden in the incubator for 20 min, after which organoid media was added to the well and cultured at 37 °C in a humidified atmosphere containing 5% CO2.

### RNA isolation and real-time RT-qPCR

RNA isolation and real-time RT-qPCR were performed as described previously^46,47^. Briefly, RNA was isolated from cells using Trizol method. The expression of the genes were analyzed using real-time RT-qPCR. After isolation, RNA concentration was measured using a Nanodrop ND-1000 system, followed by DNase treatment with DNase kit (Promega, Madison, WI, U.S.A.) and then the cDNA was synthesized using high-capacity cDNA synthesis kit (Invitrogen, Grand Island, NY, U.S.A.). Relative mRNA levels were measured with SYBR Green detection system using the QuantStudio3 real-time PCR system (Applied Biosystems, Foster City, CA, U.S.A.). All samples were tested in triplicates and the relative level of each gene was calculated by normalizing the cycle threshold (Ct) value of the gene being studied to that of the housekeeping gene 18S. Total RNA (for RNA-seq) from HPAF-II/NTC and HPAF-II/SLC38A5/CRISPR KO Clone 8 xenograft tumor samples were isolated by adding a small volume of liquid nitrogen to a mortar and pestle, then adding the previously snap-frozen tumor chunk and grinding it until a fine powder was made. Trizol was then added to this powder and transferred to a centrifuge tube. The tube was left at room temperature for 5 min and then the appropriate amount of chloroform was added, mixed, and centrifuged. The supernatant was collected and an equal volume of 70% Ethanol was added, mixed, and then added to RNA collection columns (QIAGEN, Valencia, CA, U.S.A.) and processed according to the QIAGEN protocol. Details of all primers used for the study are as shown in Table 1.

**Table 1:** Human primer sequences for real-time quantitative RT-PCR.

Protein name	Gene name	Orientation	Sequence
SN2/SNAT5	SLC38A5	Forward	AACCTGAGTCTGAGTTGCGG
		Reverse	AGGGAGGGCTCCATTCATCT
ASCT1	SLC1A4	Forward	CTCTCCTCGCCTTTCTCGCA
		Reverse	CTCTGGCAAAAGACGGGGTT
ASCT2, AAAT	SLC1A5	Forward	CTCCGCCGCCATCAACG
		Reverse	CCCCACGGGCACCTTCA
GLYT1	SLC6A9	Forward	TGGTCTCCTTCCCCAACTCA
		Reverse	CCCACGCTCGTCAGTACAAA
ATB^0,+^	SLC6A14	Forward	GAAGGAGAAAGTGTCGGCTTCA
		Reverse	TACCACCTTGCCAGACGATTTG
B^0^AT1	SLC6A19	Forward	CTCTTCACGCCCAACGTCA
		Reverse	ACCGATGTGAAGCCGTTGAT
CAT1	SLC7A1	Forward	GCGTCCGTTGGTCCTTGAG
		Reverse	AAGGTTTCAGAATCCAAGCCG
LAT1	SLC7A5	Forward	CCGTGCCGTCCCTCGTGTTC
		Reverse	GGTTCACCTTGATGGGCCGCT
y^+^LAT2	SLC7A6	Forward	CCGAGGCAGACAAGTGGAAT
		Reverse	GACAAACGGTTCCTGCACAC
y^+^LAT1	SLC7A7	Forward	ATATCCAGGACCAAGGAGGCAA
		Reverse	CACCCAAAGGGGAGGTTTCC
b^0,+^AT	SLC7A9	Forward	AGCCTGGCGTTTTACAATGG
		Reverse	AGGCAGGTTTCTGTAAGGGTT
SNAT1, ATA1	SLC38A1	Forward	TTTGGAGTCGTAGGAGTTACATCT
		Reverse	TGGAAACTGGAGGAAGAGAAAGA
SNAT2, ATA2	SLC38A2	Forward	CAAGCTGCTCTGAAAAGCCAT
		Reverse	CAAGGATTCCACTGCCCACA
SNAT3, SN1	SLC38A3	Forward	GAGGCCAGACATCTGACTGTT
		Reverse	GGTCCTCGACCCTCTGGTT
SNAT4	SLC38A4	Forward	GCGTGCACGGAGGATCG
		Reverse	CCAACTTGCGTCAGCTCTGTG
SNAT7	SLC38A7	Forward	AAAGAATCCCCCAAGCTCCATT
		Reverse	CTTCAGCAGAGCTGGAGGAAA
LAT4	SLC43A2	Forward	GTCGACCTGGAGGTGAAGTG
		Reverse	GAGGTGTAGAGGCCAACTGTC

### Plasmids and transfection

All SLC38A5 shRNA variants (TRCN0000044003, TRCN0000044004, TRCN0000044005, TRCN0000044006, TRCN0000044007), both SLC38A5 CRISPR constructs (HSPD0000127241, HSPD0000127243), and CRISPR negative control ((LV01) U6-gRNA:ef1a-puro-2A-Cas9-2A-tGFP) were purchased from Sigma-Aldrich. pLK0.1 puro (8453) was purchased from addgene. PLP1, PLP2, and VSVG (Invitrogen, K497500) were purchased from Fisher Scientific. Lipofectamine 3000 (L3000015) was purchased from ThermoFisher Scientific. For plasmid transfection, HEK293FT cells were plated to form a 50% to 60% confluent culture. For lentiviral transduction, the target cell lines were plated and grown to 60–70% confluency. The HEK293FT cells were transfected using Lipofectamine 3000, according to the ThermoFisher protocol. The lentiviral particles in media were harvested from HEK293FT cells at 48 h post transfection, filtered using 0.45 μm syringe filter, and then applied to HPAF-II cells.

For CRIPSR transduction, HPAF-II cells were plated and grown to 60–70% confluency. The viral particles (isolated and titrated by Sigma-Aldrich) were introduced at a MOI of 5 to the HPAF-II cells. For both shRNA and CRISPR transductions, the viral medium was replaced with complete medium after 18–20 h, and cells were maintained in complete medium for 24 h. After 24 h, cells underwent selection for 10–14 days with 0.5 μg/ml puromycin added to the medium. To obtain single clones, transduced cells were trypsinized and serial diluted in 96-well plates where wells with single cells were observed and selected. Thereafter, all transduced cells were maintained in complete medium with 0.5 μg/ml puromycin. Knockdown of SLC38A5 was confirmed by qPCR and [^3^H] Serine uptake assay to assess SLC38A5 mRNA expression and transport function, respectively.

### Immunofluorescence

Immunofluorescence studies for organoids were performed as previously described by Tuveson lab^48^. In short, organoids were plated in chamber slides 2–3 days prior to fixing, blocking, and then staining with rabbit anti-SLC38A5 overnight at 25 °C. Cells were then washed and stained with secondary antibodies (goat anti-rabbit) conjugated with Alexa Fluor 488, followed by washing and mounting with ProLong Diamond Antifade Mountant with DAPI. All images were captured using a Carl Zeiss LSM510 Meta upright confocal microscope.

### Colony formation assay

Colony formation assays were performed as previously described^47^. Briefly, HPAF-II/pLKO.1-PURO and HPAF-II/pLKO.1-PURO/SLC38A5/shRNA cells as well as HPAF-II/NTC and HPAF-II/SLC38A5/sgRNA KO cells were first grown in complete culture media without additives; cells were then counted and plated in 6-well plates at 1 × 10^3^ cells/well. After being allowed to form colonies for 2 weeks, cells were fixed, stained, and evaluated as previously described, using Enhanced Gram Crystal Violet (Remel).

### Animal welfare and ethical statement

Female athymic nude mice (8-week-old) were purchased from Jackson Laboratories (Bar Harbor, ME, USA). Animals were housed @ 5 mice/cage with ad libitum access to food (chow diet) and water. The room was maintained at a temperature of 22 °C with a humidity of 40–60%, and 12:12 h light/dark cycle. Mice were housed in specific pathogen-free zones. Cages were lined with sterilized corncob bedding material. Mice were given ~ 7 days to acclimatize to the housing conditions before the start of the experiments. All experimental procedures were in compliance with the National Institute of Health guidelines and approved by the TTUHSC Institutional Animal Care and Use Committee. All studies involving animals are reported in accordance with the ARRIVE guidelines for reporting experiments involving animals. Mice were not deprived of food or water at any time. Efforts were made to minimize animal suffering.

### Xenograft studies

Xenograft studies were performed as described previously^46,47^. Briefly, eight-week-old female athymic nude mice, purchased from Jackson Laboratories, were allowed to acclimatize to the environment for about a week before the start of the experiment. Mice were randomly divided into two groups for the shRNA-mediated SLC38A5 knockdown experiment as well as for the CRISPR-Cas9-mediated SLC38A5 knockout experiment. In the shRNA-mediated SLC38A5 knockdown experiment, HPAF-II/pLKO.1-PURO and HPAF-II/pLKO.1-PURO/SLC38A5/shRNA cells were subcutaneously injected at a concentration of 1 × 10^6^ cells/100 μl of serum-free media. Likewise, for the CRISPR-Cas9-mediated SLC38A5 knockout experiment, HPAF-II/NTC & HPAF-II/SLC38A5-sgRNA KO cells were subcutaneously injected at a concentration of 5 × 10^5^ cells/100 μl of serum-free media. All cells were suspended in serum-free media and matrigel (1:1 ratio). Tumor size was measured 2–3 times weekly with caliper, with tumor volume calculated using the formula (width^2^ × length)/2. The experiments were terminated 19–41 days post injection; mice were euthanized via isoflurane induction, followed by cervical dislocation and tumors harvested following the approved IACUC protocol. Following extraction, the tumor samples were weighed, divided them into smaller tumor chunks and snap-frozen in liquid nitrogen and stored at − 80 °C for downstream analysis like Real-time PCR, RNA-sequencing, Western blotting and metabolomics experiments. All murine experiments were performed in the Laboratory Animal Resource Center at Texas Tech University Health Science Center in Lubbock, TX.

### Uptake measurement

Radiolabeled uptake studies were performed as described previously^47^. SLC38A5 is a Na^+^-coupled transporter with the involvement of H^+^ movement in the opposite direction and therefore the transporter-mediated uptakes were done at pH 8.5 to create an outwardly directed H^+^ gradient across the plasma membrane. Since the transportable substrate of SLC38A5 i.e., serine, glycine and glutamine (used in the current experiment) are also transported by several Na^+^-coupled amino acid transporters, making it difficult to measure the actual functional contribution of SLC38A5, the uptake was conducted using LiCl containing buffer instead of NaCl. SLC38A5 is Li^+^ tolerant while the other amino acid transporters are not. The composition of the uptake buffer was 25 mM Tris/Hepes, pH 8.5, containing 140 mM LiCl, 5.4 mM KCl, 1.8 mM CaCl2, 0.8 mM MgSO4 and 5 mM glucose. Additionally, the amino acid substrates for SLC38A5 are also substrates for SLC7A5 (LAT1), which is a Na^+^-independent amino acid transporter; therefore, uptake via this transporter will contribute to the total uptake measured in the LiCl-containing buffer. To suppress SLC7A5-mediated uptake, the uptake buffer contained 5 mM tryptophan to compete with and block the transport of SLC38A5 substrates, which is mediated by SLC7A5. SLC38A5 does not transport tryptophan and therefore SLC38A5-mediated uptake is not affected by tryptophan. To determine the contribution of diffusion to the total uptake of amino acids, the same uptake buffer but with LiCl replaced isosmotically with *N*-methyl-d-glucamine chloride (NMDGCl) was used. The uptake was measured in 2 buffers; (i) LiCl-buffer, pH 8.5 with 5 mM tryptophan and, (ii) NMDGCl-buffer, pH 8.5 with 5 mM tryptophan. The difference in the uptake between the two buffer conditions reflects the actual SLC38A5-mediated uptake.

### RNA sequencing and analysis

The quality assessment of the raw reads was done by FastQC v0.11.7 (https://www.bioinformatics.babraham.ac.uk/projects/fastqc/) and adaptor sequences were trimmed using fastx_clipper (http://hannonlab.cshl.edu/fastx_toolkit/index.html). Reads shorter than 18 nt were removed and were sequentially mapped to human ncRNA sequences namely rRNAs, tRNAs, snRNAs, and snoRNAs downloaded from RNAcentral (https://rnacentral.org/) using Bowtie^49^ to remove reads aligning to the same. The filtered reads were mapped to human cDNA reference sequence assembly (https://ftp.ensembl.org/pub/release-109/fasta/homo_sapiens/cdna/Homo_sapiens.GRCh38.cdna.all.fa) using kallisto^50^. Differential expression analysis of target genes was done using R package DESeq2^51^.

Pathway enrichment analysis was done using Gene set enrichment analysis (GSEA) software version 4.3.2^52^. The transcript abundance from a ranked list of all available genes (whole-genome ranked list without using a cut-off) was analyzed to identify statistically significant enrichment of regulation of functionally related gene sets. Analyses in GSEA were performed on transcripts per million generated by Kallisto. The analysis used h.all.v2023.1Hs.symbols.gmt (Hallmark) and c2.cp.kegg.v2023.1.Hs.symbols.gmt (KEGG) gene set databases with the settings of perform; 1000 permutations, collapse dataset to gene symbols—false, permutation type—gene_set, enrichment statistic—weighted, metric for ranking genes—Signal2Noise, gene list sorting mode—real, gene list ordering mode—descending, max size of gene sets—500, and min size of gene sets—15.

### Metabolomic analysis

Xenograft tumors from HPAF-II/NTC (n = 10) and HPAF-II/SLC38A5-sgRNA KO Clone 8 (n = 10) were used for metabolomic analysis (UCDavis West Coast Metabolomic Center). Samples were extracted using 1 ml of 3:3:2 ACN:IPA:H2O (v/v/v). Half of the sample was dried to completeness and then derivatized using 10 μl of 40 mg/ml of Methoxyamine in pyridine. They were then shaken at 30 °C for 1.5 h. Then 91 μl of MSTFA + FAMEs were added to each sample and were shaken at 37 °C for 0.5 h to finish derivatization. Samples were then vialed, capped, and injected onto the instrument. A 7890A GC coupled with a LECO TOF was used. 0.5 μl of derivatized sample was injected using a splitless method onto a RESTEK RTX-5SIL MS column with an Intergra-Guard at 275 °C with a helium flow of 1 ml/min. The GC oven was set to hold at 50 °C for 1 min then ramp to 20 °C/min to 330 °C and then held for 5 min. The transfer line was set to 280 °C while the EI ion source was set to 250 °C. The Mass spec parameters collect data from 85 to 500 m/z at an acquisition rate of 17 spectra/s. After data curation, the data is then normalized using sum normalization using the sum of the metabolites (mtic). The raw peak height for metabolite *i* of sample *j* is divided by the sum of the identified metabolites (mtic) of sample j then multiplied by the average sum of identified metabolites of the samples in the study (mtic average). Prior to further metabolomic data analysis, the identified metabolite data were pre-processed. The raw metabolite list with peak intensity was used as input using MetaboAnalyst 4.0 (http://www.metaboanalyst.ca), the online platform intended for metabolomic analysis. The heatmap of the metabolites (amino acid) between the NTC and SLC38A5/CRISPR KO tumors was generated using Graphpad Prism 9 Software.

### Mitochondrial respiration assays

OCR (oxygen consumption rate) and ECAR (extracellular acidification rates) measurements were performed using the Seahorse XFe96 extracellular flux analyzer (Seahorse, Agilent Technologies) and the Seahorse XF Cell Mito Stress Test kit. The Cell Mito Stress Test kit contains oligomycin, carbonyl cyanide-4-(trifluoromethoxy) phenylhydrazone (FCCP), and rotenone/antimycin A. Briefly, NTC and SLC38A5/CRISPR KO cells were seeded at 2 × 10^4^/well in their growth medium. The plate was kept in a cell culture hood for 1 h at room temperature to allow cells to promote uniform adherence on the well bottoms and decrease edge effects. Then the cells were incubated overnight in a humidified 37 °C incubator at 5% CO_2_. Meantime, the sensory cartridge and utility plate were detached, while the sensory cartridge was placed upside down, 200 µl Seahorse XF Calibrant was added to all wells in the utility plate, and the sensory cartridge was softly lowered back onto the utility plate to avoid creating bubbles. Then, the sensory cartridge was also incubated overnight in a non-CO_2_ incubator at 37 °C. Prior to performing the assay, growth medium in the wells were exchanged with the assay medium (XF DMEM, Agilent Technologies) supplemented with 10 mM glucose, 1 mM pyruvate and 2 mM glutamine. Then, the plate was incubated in a non-CO_2_ incubator at 37 °C for 45 min prior to the start of the assay. Meantime, oligomycin (2 µM), FCCP (1 µM), and rotenone and antimycin A (0.5 µM) were added in port A, B, and C of the sensory cartridge, respectively. Then both utility plate and sensory cartridge were placed in the Seahorse instrument for calibration. After the calibration, the utility plate was exchanged with the cell plate to obtain the OCR and ECAR measurements. OCR and ECAR data points refer to the average rates during the measurement cycles. OCR were reported as “pmoles/min”, and ECAR as “mpH/min”. Three baseline measurements were taken prior to the addition of any compound, and 3 measurements were taken after the addition of each compound. After measurements were complete, the results were automatically analyzed by the wave software, and the data were exported as Excel. Results are presented as mean ± SEM from six to eight replicate wells.

### Statistical analysis

Statistical analysis and graphs were performed using GraphPad Prism 8.4.3. Results are expressed as mean ± SEM. All experiments were repeated thrice unless otherwise specified. Statistical significance was determined using unpaired Student’s t-test and *p* values indicated as follows: NS, non-significant, *p < 0.05, **p < 0.01, ***p < 0.001, ****p < 0.0001.

### Human subjects

No human participants were directly involved in our study. The organoids (normal and tumor) were developed by the Tuveson lab and were procured after completing the Materials Transfer Agreement. The PDXs are available at the TTUHSC Cancer Center. This core laboratory serves as the Cell Culture and Xenograft Repository for the Children’s Oncology Group and also is the Texas Cancer Cell Repository (www.TXCCR.org). New cell lines and xenografts are initiated from both adults and children with cancer, expanded, characterized, and readied for distribution. This repository contains a huge collection of patient-derived xenograft tissues and cell lines from patients with pancreatic cancer; these resources are readily available to the Principal Investigator for use after completing the Materials Transfer Agreement.

### Supplementary Information

Supplementary Figures.

## Data Availability

The raw sequence data reported in this paper have been deposited in the Genome Sequence Archive (Genomics, Proteomics & Bioinformatics 2017) in BIG Data Center (Nucleic Acids Res 2017), Beijing Institute of Genomics (BIG), Chinese Academy of Sciences^53,54^, under accession number PRJCA018857, HRA005223 that are publicly accessible at https://download.cncb.ac.cn/gsa-human/HRA005223.

## Abbreviations

PDAC: Pancreatic ductal adenocarcinoma
PNET: Pancreatic neuroendocrine tumor
OXPHOS: Oxidative phosphorylation
TCA: Tricarboxylic acid cycle
mTORC1: Mammalian target of rapamycin 1
TCGA: The Cancer Genome Atlas
NMDG: *N*-Methyl-d-glucamine
NTC: Non-targeted control
IPA: Ingenuity pathway analysis
NADH: Nicotinamide adenine dinucleotide (reduced)
FADH_2_: Flavin adenine dinucleotide (reduced)
