# Supplementary material for: Amino acid transporter SLC38A5 is a tumor promoter and a novel therapeutic target for pancreatic cancer
Source: Sci Rep. 2023 Oct 6;13:16863. doi: 10.1038/s41598-023-43983-1 (PMC10558479; doi:10.1038/s41598-023-43983-1)

Supplemental Information

Amino acid transporter SLC38A5 is a tumor promoter and a novel therapeutic target for pancreatic cancer

Tyler Sniegowski[1], Devaraja Rajasekaran[1], Souad R. Sennoune[1], Sukumaran Sunitha[2], Fang Chen[2], Mohamed Fokar[2], Sudhir Kshirsagar[3], P. Hemachandra Reddy[3], Ksenija Korac[1], Mosharaf Mahmud Syed [1], Tanima Sharker[1], Vadivel Ganapathy[1], Yangzom D. Bhutia[1*]

[1]Department of Cell Biology and Biochemistry, Texas Tech University Health Sciences Center, Lubbock, TX 79430

[2]Center for Biotechnology & Genomics, Texas Tech University, Lubbock TX 79409

[3]Department of Internal Medicine, Texas Tech University Health Sciences Center, Lubbock, TX 79430

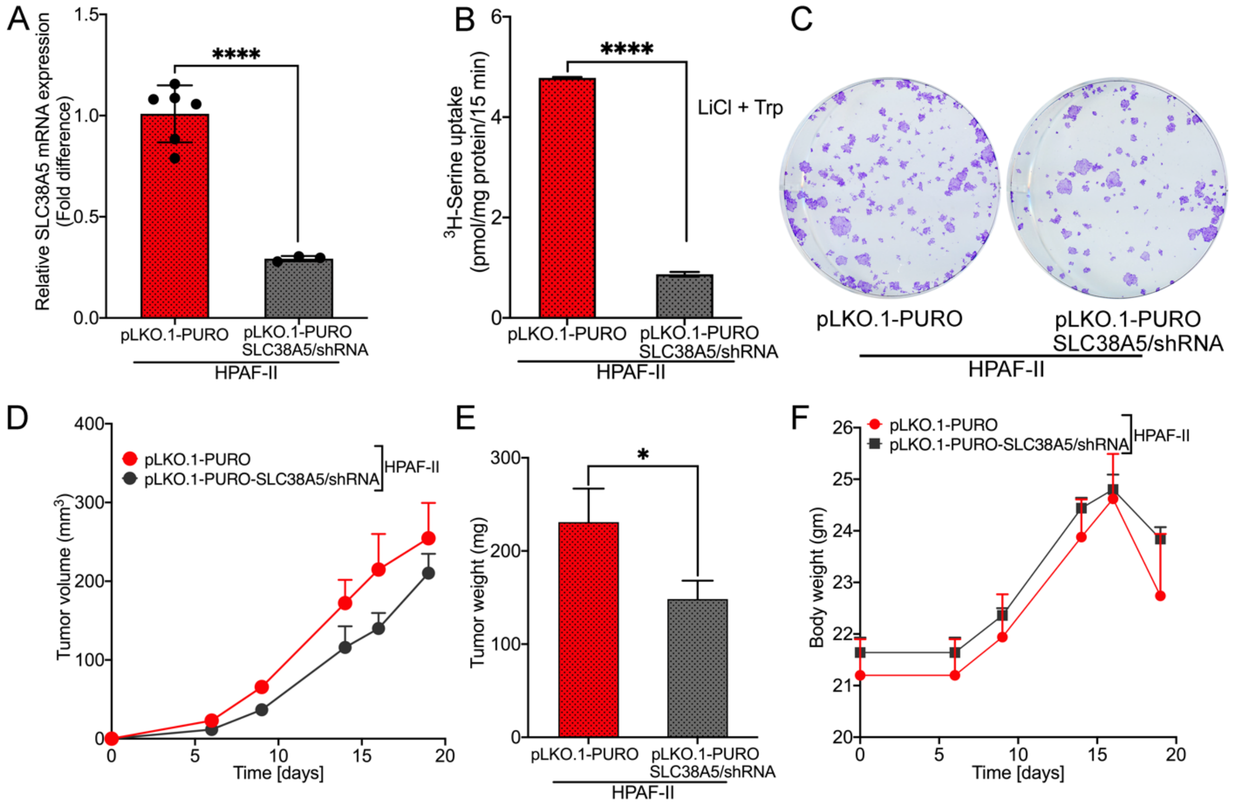

Figure S1

Figure S1. shRNA-mediated knockdown of SLC38A5 indicates the tumor promoting ability of SLC38A5. (A) Real-time PCR showing SLC38A5 mRNA expression in HPAF-II/pLKO.1-PURO and HPAF-II/pLKO.1-PURO/SLC38A5/shRNA cell lines. (B) [^{3}H]-Serine uptake in HPAF-II/pLKO.1-PURO versus HPAF-II/SLC38A5/shRNA knockdown cells in LiCl buffer containing 2.5 mM tryptophan at pH 8.5. (C) Colony formation assay in HPAF-II/pLKO.1-PURO and HPAF-II/SLC38A5/shRNA knockdown cell lines. (D) Subcutaneous xenograft study showing tumor volume, (E) Tumor weight, and (F) Body weight in athymic nude mice implanted with HPAF-II/pLKO.1-PURO and HPAF-II/pLKO.1-PURO/SLC38A5/shRNA cell lines. Data are given as mean ± SEM. *$p<0.05$, ****$p<0.0001$.

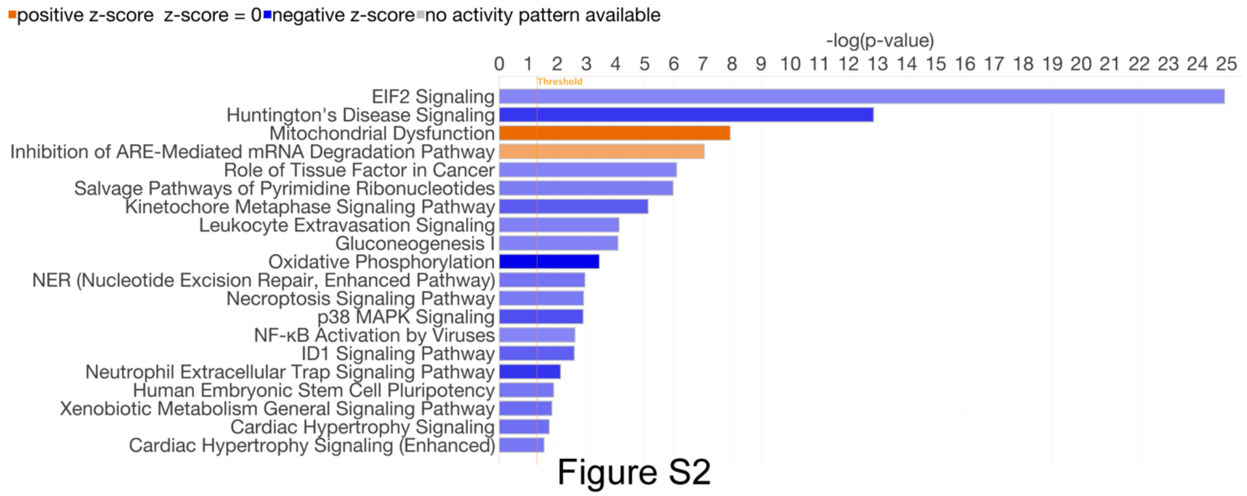

Figure S2

Figure S2. Ingenuity Pathway Analysis using RNA sequencing data between NTC tumors and SLC38A5/CRISPR KO Tumors. (A) Ingenuity Pathway Analysis plot showing top 20 enriched canonical pathways as analyzed by the IPA software. Positive z-score shown in orange identifies activated pathway and negative z-score in blue color specifies inhibited pathways after CRISPR-mediated knockout of SLC38A5.

A

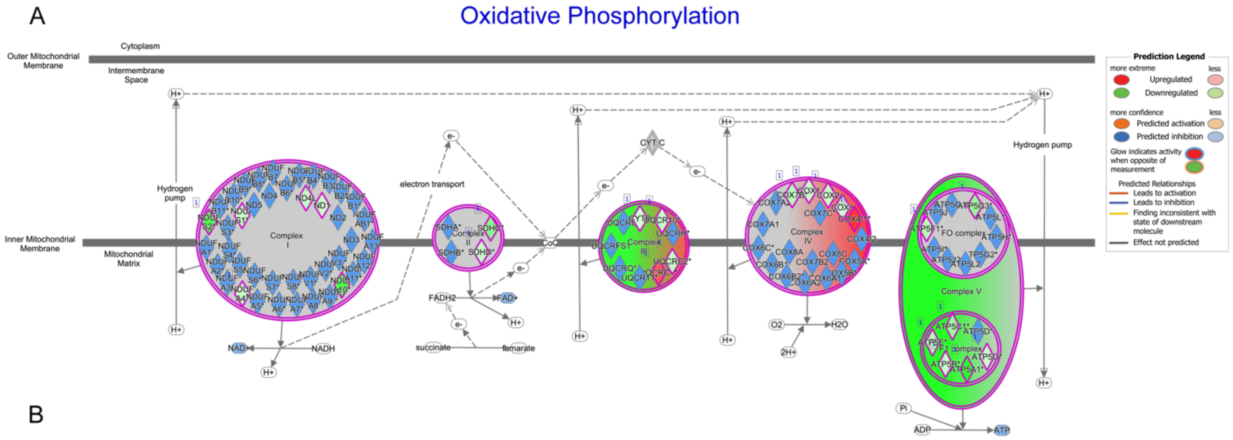

B

Symbol	Entrez Gene Name	Expr Log Ratio	Expr p-value	Expected	Location	Type(s)
ATP5F1A	ATP synthase F1 subunit alpha	-9.866	3.49E-10	Up	Cytoplasm	transporter
ATP5F1B	ATP synthase F1 subunit beta	-2.409	1.1E-14	Up	Cytoplasm	transporter
ATP5F1C	ATP synthase F1 subunit gamma	-4.379	0.000000162	Up	Cytoplasm	transporter
ATP5F1E	ATP synthase F1 subunit epsilon	-3.187	4.67E-15	Up	Cytoplasm	transporter
ATP5MC3	ATP synthase membrane subunit c locus 3	-4.835	6.75E-08	Up	Cytoplasm	transporter
ATP5PB	ATP synthase peripheral stalk-membrane subunit b	-3.325	0.000000254	Up	Cytoplasm	transporter
ATP5PO	ATP synthase peripheral stalk subunit OSCP	-2.204	0.000236	Up	Cytoplasm	transporter
COX4I1	cytochrome c oxidase subunit 4I1	10.635	1.24E-13	Up	Cytoplasm	enzyme
COX7A2L	cytochrome c oxidase subunit 7A2 like	-2.263	0.00685	Up	Cytoplasm	enzyme
COX7B	cytochrome c oxidase subunit 7B	-3.334	4.09E-09	Up	Cytoplasm	enzyme
CYB5B	cytochrome b5 type B	-9.091	8.06E-09		Cytoplasm	enzyme
DMAC2L	distal membrane arm assembly component 2 like	-5.906	0.00806	Up	Cytoplasm	transporter
MT-CO1	cytochrome c oxidase subunit I	-2.556	8.12E-09	Up	Cytoplasm	enzyme
MT-CO2	cytochrome c oxidase subunit II	-2.555	1.27E-09	Up	Cytoplasm	enzyme
MT-CO3	cytochrome c oxidase subunit III	-2.313	2.37E-09	Up	Cytoplasm	enzyme
MT-CYB	cytochrome b	-2.08	0.0000112	Up	Cytoplasm	enzyme
MT-ND1	NADH dehydrogenase subunit 1	-2.325	0.000000151	Up	Cytoplasm	enzyme
MT-ND4L	NADH dehydrogenase subunit 4L	-2.63	7.32E-09	Up	Cytoplasm	enzyme
NDUFA4	NDUFA4 mitochondrial complex associated	-2.48	0.00017	Up	Cytoplasm	enzyme
NDUFA10	NADH:ubiquinone oxidoreductase subunit A10	-8.476	0.000000886	Up	Cytoplasm	transporter
NDUFS1	NADH:ubiquinone oxidoreductase core subunit S1	-2.821	0.00101	Up	Cytoplasm	enzyme
NDUFS2	NADH:ubiquinone oxidoreductase core subunit S2	-8.307	0.000000164	Up	Cytoplasm	enzyme
SDHC	succinate dehydrogenase complex subunit C	-5.469	0.00324	Up	Cytoplasm	enzyme
SDHD	succinate dehydrogenase complex subunit D	-2.548	0.00107	Up	Cytoplasm	enzyme
UQCR10	ubiquinol-cytochrome c reductase, complex III subunit X	-2.55	0.000169	Up	Cytoplasm	enzyme
UQCRC1	ubiquinol-cytochrome c reductase core protein 1	7.893	0.000000673	Up	Cytoplasm	enzyme
UQCRC2	ubiquinol-cytochrome c reductase core protein 2	-2.864	0.00164	Up	Cytoplasm	enzyme

Figure S3

Figure S3. Oxidative phosphorylation pathway and molecules associated with it. (A) Oxidative phosphorylation pathway showing changes with the associated genes in response to SLC38A5 knockout. (B). Table showing 27 genes associated with oxidative phosphorylation that are downregulated in response to SLC38A5 knockout.

Full-size blots

Figure 6A

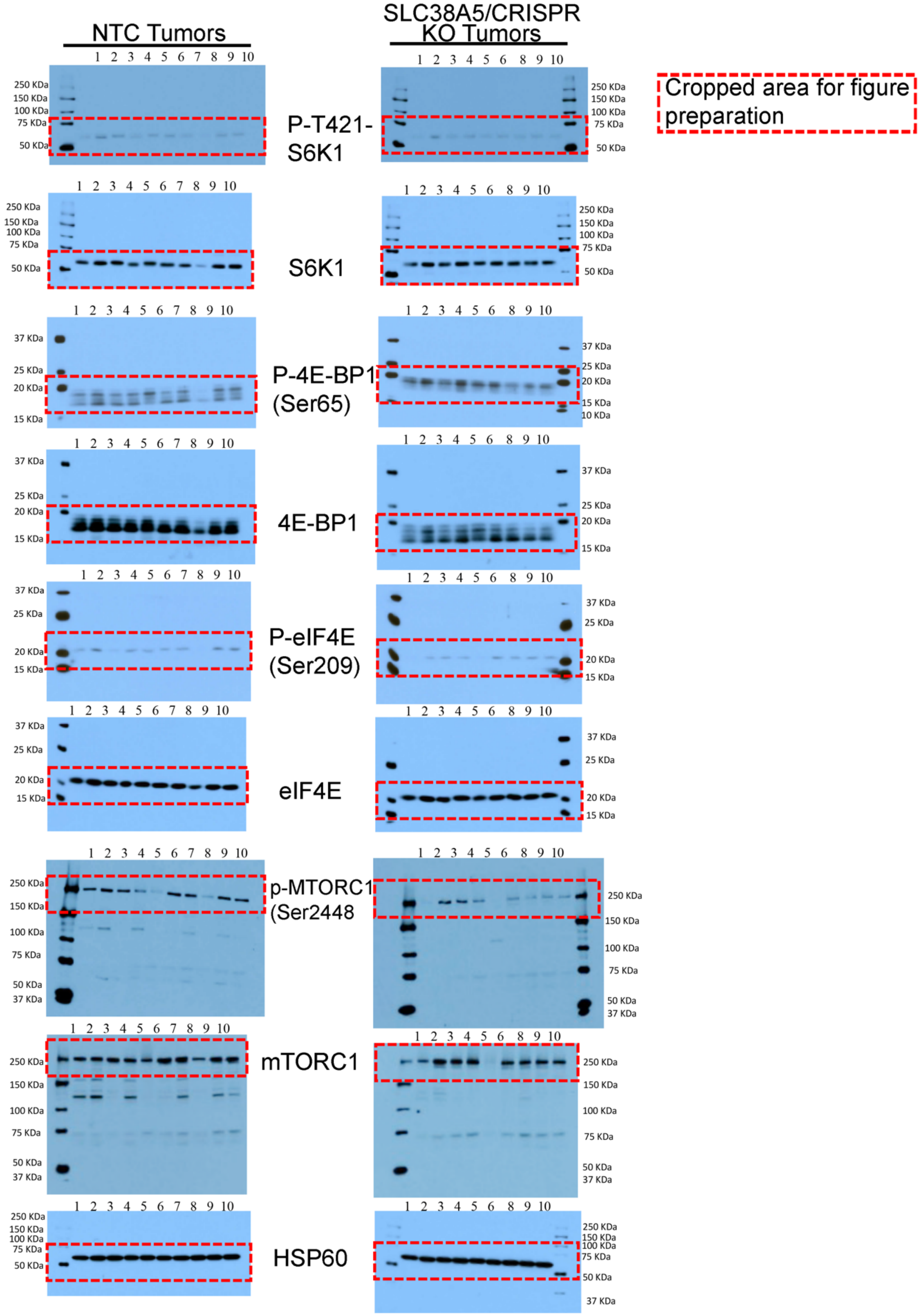
NTC Tumors
SLC38A5/CRISPR KO Tumors
Cropped area for figure preparation
P-T421-S6K1
S6K1
P-4E-BP1 (Ser65)
4E-BP1
P-eIF4E (Ser209)
eIF4E
p-MTORC1 (Ser2448
mTORC1
HSP60
1 2 3 4 5 6 7 8 9 10
1 2 3 4 5 6 8 9 10
250 KDa
150 KDa
100 KDa
75 KDa
50 KDa
37 KDa
25 KDa
20 KDa
15 KDa
10 KDa

Figure 7J: HSP 60

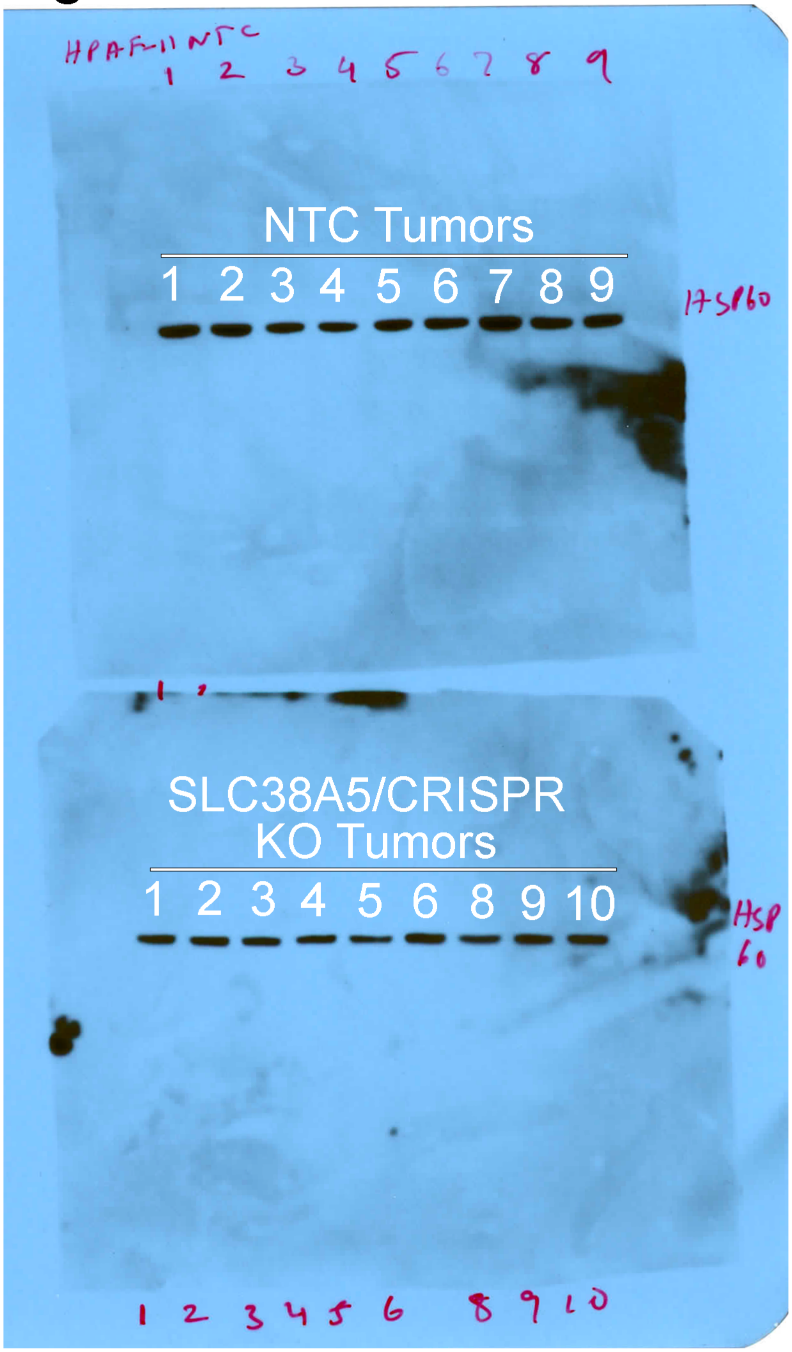

Supplement: Supplementary file 1 — Supplementary Figures. [file 41598_2023_43983_MOESM1_ESM.pdf]
